# Two Modes of Transcriptional Activation at Native Promoters by NF-κB p65

**DOI:** 10.1371/journal.pbio.1000073

**Published:** 2009-03-31

**Authors:** Dominic van Essen, Bettina Engist, Gioacchino Natoli, Simona Saccani

**Affiliations:** 1 Max Planck Institute for Immunobiology, Freiburg, Germany; 2 Department of Experimental Oncology, European Institute of Oncology (IEO), IFOM-IEO Campus, Milan, Italy; National Cancer Institute, United States of America

## Abstract

The NF-κB family of transcription factors is crucial for the expression of multiple genes involved in cell survival, proliferation, differentiation, and inflammation. The molecular basis by which NF-κB activates endogenous promoters is largely unknown, but it seems likely that it should include the means to tailor transcriptional output to match the wide functional range of its target genes. To dissect NF-κB–driven transcription at native promoters, we disrupted the interaction between NF-κB p65 and the Mediator complex. We found that expression of many endogenous NF-κB target genes depends on direct contact between p65 and Mediator, and that this occurs through the Trap-80 subunit and the TA1 and TA2 regions of p65. Unexpectedly, however, a subset of p65-dependent genes are transcribed normally even when the interaction of p65 with Mediator is abolished. Moreover, a mutant form of p65 lacking all transcription activation domains previously identified in vitro can still activate such promoters in vivo. We found that without p65, native NF-κB target promoters cannot be bound by secondary transcription factors. Artificial recruitment of a secondary transcription factor was able to restore transcription of an otherwise NF-κB–dependent target gene in the absence of p65, showing that the control of promoter occupancy constitutes a second, independent mode of transcriptional activation by p65. This mode enables a subset of promoters to utilize a wide choice of transcription factors, with the potential to regulate their expression accordingly, whilst remaining dependent for their activation on NF-κB.

## Author Summary

Transcriptional activation by the NF-κB family of transcription factors is crucial for the expression of multiple genes involved in cell survival, proliferation, differentiation, and inflammation. The activation domain of the p65 subunit of NF-κB has been extensively studied in vitro and on artificial reporter plasmids, but the molecular basis by which it drives expression of natural target genes in vivo is still not well understood. Moreover, it is unclear how any single activation mechanism could allow different target genes to fine tune their timing and expression according to their biological requirements. To address this, we experimentally blocked the interaction of p65 with the Mediator complex—a key factor for transcription by most, if not all, activators. While this prevented expression of many NF-κB–dependent genes, others were unaffected, revealing that p65 is able to drive their expression by an independent mode, which does not depend on direct contact with Mediator. Further experiments indicated that p65 accomplishes this by controlling the recruitment of other, secondary transcription factors to its target promoters. This may enable NF-κB to retain overall control over activation of its target genes, but at the same time allow secondary transcription factors to specify appropriate expression levels according to the cell-type and stimulus.

## Abstract 3

The p65 subunit of NF-κB drives expression of target genes not only as a classical activator, via direct interactions with the basic transcriptional machinery, but also independently by controlling the recruitment of secondary transcription factors to target promoters.

## Introduction

The goal of understanding transcriptional activation encompasses the description of an unbroken chain of events leading from the binding of a transcription factor to its natural target promoters in an intact cell, until the initiation of mRNA synthesis by RNA polymerase II (pol-II). In the case of the NF-κB family of transcription factors, this is a challenging task, since the tremendous functional diversity of its target genes makes it difficult to imagine a single activation mechanism able to satisfy the needs of all of them.

Transcription factors belonging to the NF-κB family are found in metazoan organisms ranging from insects to mammals, and are essential in regulating the activation of hundreds of genes in response to various extracellular stimuli and developmental cues [1]. In most vertebrate cell types, NF-κB exists as a combination of five related proteins: p65, c-Rel, RelB, p50, and p52. They share a structurally conserved Rel homology region at their amino terminus, which is responsible for dimerization, interaction with inhibitory IκB proteins, nuclear entry, and binding to their specific DNA target sequences (known as κB sites). In unstimulated cells, dimers of NF-κB are held in the cytoplasm through the binding of inhibitory proteins (IκBs or p100), but upon stimulation they are released to enter the nucleus. There they are capable of binding with high affinity to their target sequences, found both in gene promoters and in enhancer regions [2]. In contrast to our detailed understanding of the signalling events that control the level of NF-κB present in the nucleus, little is known about the mechanisms of transcriptional activation by the various dimer species whilst bound to endogenous target genes. It is particularly unclear whether promoter binding by a given NF-κB dimer always triggers the same fixed response, leading to an identical transcriptional output at all genes, or, as seems more reasonable, different genes should somehow be able to fine tune their transcription levels after binding and activation by NF-κB. However, the transcriptional activation domain of p65 has been extensively studied in vitro and on artificial reporter plasmids, and the data from these systems provide a foundation on which one can try to build an understanding of its function on natural promoters.

During the last two decades, experiments using reconstituted cell-free systems have succeeded in defining the minimal apparatus needed to drive activated transcription. An essential set of general transcription factors (GTFs) is sufficient to direct the binding of, and initiation of basal transcription by pol-II at the core regions of most promoters [3]. In order to respond to transcriptional activators such as NF-κB, though, additional elements are required, foremost amongst which is the Mediator complex. This is a large, multi-subunit complex, which was independently identified by several laboratories through its ability to bind to various transcriptional activation domains (including that of p65), or by its necessity as a co-factor for transcriptional activation in vitro by other transcription factors (reviewed by Malik and Roeder [4]). Using highly purified components in vitro, the combination of pol-II, GTFs, and the Mediator complex is sufficient to drive transcriptional activation by NF-κB [5]. Conversely, depletion of Mediator from total nuclear extracts using antibodies abolishes all in vitro transcription by pol-II, including the response to activators such as p65 [6,7].

The capacity of p65 to activate transcription has also been the subject of numerous studies using synthetic reporter plasmids in transfected cell lines. In this context, the carboxy terminus of p65 (like those of c-Rel and RelB) is able to drive transcription in isolation when fused to a heterologous DNA-binding domain, leading to its definition as a transcriptional activation domain (TAD [8,9]). Since the Mediator complex has been shown to interact with the TAD of p65 [10], a straightforward model would be that direct binding to Mediator constitutes the initial, essential step in p65-driven transcription; however, to our knowledge this has never been tested in vivo at native promoters. Part of the reason for this may be that the Mediator complex is essential for viability, and thus it is not readily amenable to loss-of-function-based experiments in intact cells. In order to test the requirement for this interaction at native NF-κB target promoters, we sought to disrupt the contact between p65 and Mediator by eliminating a single Mediator subunit in vivo.

We found that contact with Mediator is indeed essential for p65 to drive the expression of many NF-κB target genes. Unexpectedly, though, many others were still expressed normally even when this contact was disrupted. Further experiments revealed that p65 has a second, independent mode of transcriptional activation, which acts by regulating promoter occupancy by secondary transcription factors.

## Results

### Removal of Trap-80 Disrupts the Interaction of p65 with Mediator

We wanted to identify a subunit of the Mediator complex that directly contacts p65, and whose removal would abolish the interaction of p65 with the remaining complex. As a cue, we noted that the *Drosophila* NF-κB homologue Dif has been shown to interact with Med17 (amongst other Mediator subunits [11]). Although the TAD of p65 shows no obvious sequence homology with that of Dif, we speculated that it might nonetheless contact the corresponding mammalian Mediator subunit, Trap-80. Using the yeast two-hybrid system, we were able to detect an interaction between the amino-terminus of Trap-80 and the far carboxy-terminus of p65 (Figure S1A). Since none of the known components of the Mediator complex are well conserved between yeast and mammals at the primary amino acid sequence level [12], this strongly suggests that the interaction of p65 with Trap-80 is direct; however, at this point we could not exclude that it may be bridged or stabilized by interaction with some endogenous yeast protein(s).

An over-expressed, tagged form of Trap-80 could be co-immunoprecipitated with p65 from nuclear extracts of transfected HEK-293 cells (Figure S1B), confirming that the two proteins can associate into a complex together. To establish whether they occupy adjacent positions within the complex, we used the bimolecular fluorescence complementation (BiFC) approach [13]. We co-expressed fusion proteins of p65 joined, via a short peptide linker, to an amino-terminal fragment of the fluorescent protein Venus, and of Trap-80 similarly joined to a complementary fragment from the Venus carboxy-terminus. Neither of these fragments is itself fluorescent, but if brought sufficiently close by an interaction between their respective fusion partners, they form a bimolecular fluorescent entity (the maximum permissible distance separating the tethered ends is limited by the peptide linkers (16 amino acids, or around 60 Å each), and has been empirically estimated at around 100 Å [13]—roughly comparable to the diameter of the Rel homology region of p65 [14]). Fusions of Venus fragments to the carboxy-terminus of p65, or to the amino-terminus of Trap-80 were nonfluorescent when expressed alone, but, in close agreement with the yeast two-hybrid experiments, cells co-expressing both together were fluorescent, indicating that the two proteins are juxtaposed in vivo (Figure S1C).

Together, these data suggested that Trap-80 forms part of the contact surface of the Mediator complex through which it interacts with p65. Although this interaction may include regions of contact with other Mediator subunits, we considered that Trap-80 was a good candidate as a subunit whose removal might destabilize binding by p65. Therefore, we attempted to disrupt the p65-Mediator interaction by generating cell lines in which Trap-80 expression was stably knocked-down by RNA interference. At the outset, this seemed a risky approach, since in other systems Trap-80 has been shown to be essential for cell viability. Yeast with a null mutation of the homologous *Srb4* gene are nonviable, and in cells carrying a temperature-sensitive allele, most mRNA synthesis ceases at the restrictive temperature [15,16]. Likewise, dTrap-80 is needed for both basal and activated transcription in *Drosophila* SL2 cells [11], and Boube et al. [17] have shown in the *Drosophila* epidermis that mutation of dTrap-80 is lethal for cells.

**Figure 1:**
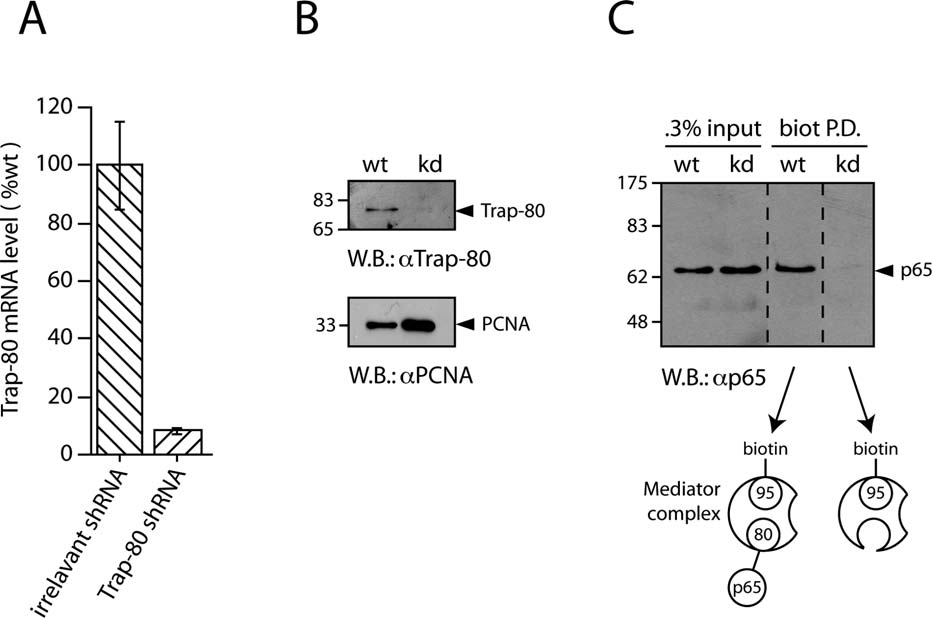
Removal of Trap-80 Disrupts the Interaction of p65 with Mediator (A) Knock-down of Trap-80 mRNA by RNA interference. Expression level of *Trap-80* mRNA in 3T3 fibroblasts expressing an shRNA targeting Trap-80, shown as a percentage of the level in control cells (expressing an irrelevant shRNA, hereafter referred to as wild type). mRNA levels were measured by quantitative PCR and normalized with respect to *Tbp*. Error bars indicate standard errors; the results presented here are representative of more than ten experiments. (B) Knock-down of Trap-80 protein levels. Nuclear extracts were prepared from wild-type (wt) or Trap-80 knock-down (kd) fibroblasts, and endogenous Trap-80 (upper panel) or PCNA (as a loading control, lower panel) was detected by western blotting. (C) Co-precipitation of p65 and Trap-95 depends on the presence of Trap-80. Wild-type and Trap-80 knock-down fibroblasts were simultaneously transduced with retroviruses expressing biotin-tagged Trap-95 and the BirA biotin ligase, and nuclear extracts were prepared after stimulation with TNF-α. The Mediator complex, containing biotinylated Trap-95, was pulled down using streptavidin beads (biot P.D.), and any associated p65 was detected by western blotting. Pull-down using in vivo biotinylation is our method of choice for analysis of proteins bound to the endogenous Mediator complex, due to the extremely high affinity of the streptavidin-biotin interaction. The levels of biotin-tagged Trap-95 were similar in both cell types, as measured by the level of co-expressed Tomato fluorescent protein (Figure S3). Dotted lines indicate where nonrelevant lanes have been cropped from the figure.

**Figure 2:**
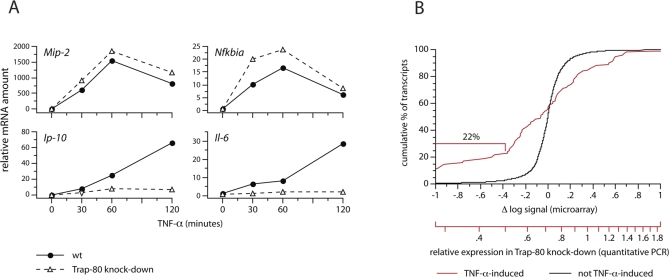
Trap-80–Dependent and –Independent Genes (A) Expression of *Mip-2*, *Nfkbia*, *Ip-10*, and *Il-6* mRNA in subconfluent cultures of wild-type and Trap-80 knock-down fibroblasts, after stimulation with TNF-α. mRNA levels are expressed relative to unstimulated, wild-type cells; the results presented here are representative of more than ten experiments. (B) Trap-80–dependence of TNF-α–induced versus noninduced genes. Cumulative percentage of transcripts whose expression in Trap-80 knock-down cells exceeds the indicated level in wild-type cells, expressed both as the Δ log signal of the microarray probes (upper scale) and the calculated relative expression level using a standard curve generated by quantitative PCR of a subset of 36 genes (lower scale). The red line represents the top 100 TNF-α–induced transcripts; the black line represents 5,000 transcripts whose levels were unchanged by TNF-α stimulation. Table S1 lists the top 50 Trap-80–dependent and –independent TNF-α–induced genes.

Strikingly, then, we were able to generate clonal lines of mouse 3T3 fibroblasts in which *Trap-80* mRNA expression was reduced by >90% compared to wild-type levels (Figure 1A), and Trap-80 protein levels were no longer detectable by western blotting (Figure 1B). These Trap-80–deficient fibroblasts proliferated equivalently to control cells and appeared morphologically normal (Figure S2), could be grown in culture for at least 12 wk, and expanded by at least 10^20^-fold (∼30 passages; unpublished data). Moreover, microarray analysis indicated that the expression levels of >96% of transcripts were changed by less than 1.5-fold in Trap-80–deficient cells (see Figure 2B later).

We used the Trap-80–deficient cells to determine whether Trap-80 is indeed essential for binding of p65 to the Mediator complex. To this end, we tested whether p65 could be co-precipitated with an alternative Mediator subunit, Trap-95, from nuclear extracts of Trap-80–deficient fibroblasts. We used streptavidin beads to pull-down the Mediator complex from cells expressing a biotin-tagged allele of Trap-95. In cells containing Trap-80, p65 was pulled-down with the Mediator complex, reconfirming their in vivo interaction (Figure 1C). However, no p65 was pulled-down from Trap-80 knock-down cells, indicating that the Trap-80 subunit is required for the interaction between p65 and Mediator in vivo.

Therefore, Trap-80–deficient cells represent an experimental system with which we could test the importance of the interaction with the Mediator complex for transcriptional activation in vivo by p65.

### Trap-80–Dependent and –Independent NF-κB Target Genes

We examined the expression of endogenous NF-κB target genes in Trap-80–deficient cells, in response to stimulation with the cytokine tumour necrosis factor-α (TNF-α). 3T3 fibroblasts are a particularly useful model system in which to study activation by p65, since in these cells most NF-κB–driven transcription relies on this subunit [18,19]. We predicted that if transcriptional activation of endogenous genes by p65 depends on its interaction with Mediator, as implied by in vitro studies [6,10], then they should not be expressed in Trap-80–deficient cells. In agreement with this, we found that expression of the *Ip-10* and *Il-6* genes was abolished in cells lacking Trap-80 (Figure 2A). Two independent small hairpin RNAs (shRNAs) targeting Trap-80 gave the same result, and expression could be restored by reconstitution of Trap-80 knock-down cells with an shRNA-resistant form of Trap-80, ruling out the possibility that the block in expression was caused by an off-target effect of the shRNAs (Figure S4).

Unexpectedly, however, two other NF-κB target genes, *Mip-2* and *Nfkbia*, were unaffected by the absence of Trap-80, and were expressed in cells containing either shRNA at the same level as in control cells (Figure 2A). To verify that transcription of these genes was indeed dependent on p65, we analysed their expression in fibroblasts derived from p65-knockout mice. In agreement with earlier published results [18,19], production of *Mip-2*, *Nfkbia*, and *Ip-10* mRNA was completely abolished, and *Il-6* mRNA levels were strongly reduced (Figure S5). Thus, in 3T3 fibroblasts, p65-dependent genes can be subdivided, depending on whether they require the interaction of p65 with the Mediator complex for their expression (Trap-80–dependent; exemplified by *Ip-10* and *Il-6*), or instead can be expressed even when this interaction is disrupted (Trap-80–independent; exemplified by *Mip-2* and *Nfkbia*).

To examine the generality of this grouping, we performed a microarray analysis of the levels of 29,000 transcripts in wild-type and Trap-80 knock-down cells, before and after stimulation of NF-κB activity using TNF-α. Genes induced by TNF-α are dominated by known NF-κB targets, and their promoters are significantly enriched for NF-κB binding motifs (Tables S1 and S2). Amongst these TNF-α–induced genes, Trap-80–dependent genes are strongly enriched (22%, compared with <3% of non-TNF-α–induced genes; Figure 2B)—supporting the importance of the interaction with Mediator for p65-driven transcription. On the other hand, when considering all Trap-80–dependent genes, although TNF-α–induced genes are significantly over-represented (10%, compared with <0.2% of Trap-80–independent genes; Figure S6), the majority are unaffected by TNF-α treatment, indicating that NF-κB is not alone in its functional requirement for Trap-80. A subset of both Trap-80–dependent and Trap-80–independent NF-κB target genes were validated by quantitative reverse transcription (RT)-PCR (Figure S7), and the results closely correlated with those of the microarray (*r =* 0.85). We chose to focus on *Ip-10*, *Il-6*, *Mip-2*, and *Nfkbia* for further study, since these displayed clear-cut dependencies on Trap-80.

### Interaction of p65 with Mediator Drives Recruitment of pol-II

**Figure 3:**
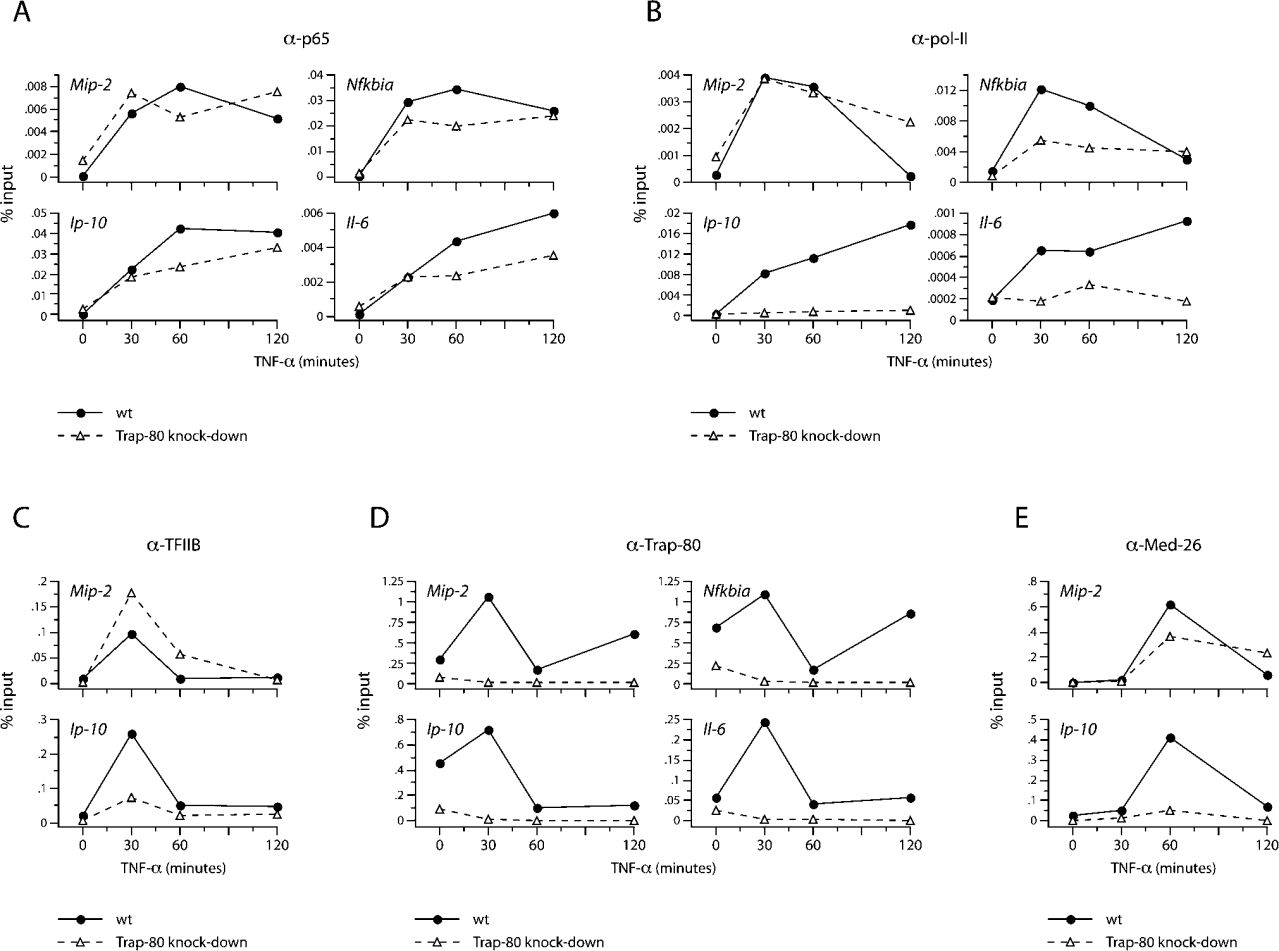
Interaction of p65 with Mediator Drives Recruitment of pol-II (A) Trap-80 knock-down does not prevent p65 recruitment to its target promoters. (B) Trap-80 knock-down blocks pol-II recruitment to Trap-80 dependent promoters. (C) Trap-80 knock-down blocks TFIIB recruitment to Trap-80 dependent promoters. (D) Presence of Trap-80 at promoter regions. (E) Trap-80 knock-down blocks Med-26 recruitment to Trap-80 dependent promoters. ChIP using antibodies against p65 (A), pol-II (B), TFIIB (C), Trap-80 (D), and Med-26 (E), after stimulation with TNF-α. Bound DNA was analyzed using primers and probes specific for promoter regions. The amounts are presented as the percentage recovered out of the total input DNA (percent input). All measurements were performed in duplicate; the results presented here are representative of two to five experiments.

We initially considered the mechanism of activation of Trap-80–dependent genes. First, we performed chromatin immunoprecipitation (ChIP) using antibodies against p65, to establish whether disrupting its interaction with Mediator could somehow inhibit p65 from binding to some of its target promoters. We found that p65 was efficiently recruited to the promoters of the Trap-80–dependent genes *Ip-10* and *Il-6* upon TNF-α stimulation, and its level of binding was only slightly reduced in Trap-80–deficient compared to wild-type cells (Figure 3A). Moreover, the level of p65 binding to the Trap-80–independent *Nfkbia* promoter was also slightly reduced to a similar extent, arguing that this is not sufficient to explain the failure in *Ip-10* and *Il-6* transcription. Binding to the *Mip-2* promoter was completely unaffected.

Next, we did ChIP with antibodies against pol-II to investigate whether its recruitment to promoters was a consequence of the p65-Mediator interaction. Indeed, association of pol-II with the promoters of *Ip-10* and *Il-6* was completely prevented in Trap-80–deficient cells (Figure 3B). In contrast, it was strongly recruited to the *Mip-2* promoter both with and without Trap-80. Pol-II was also recruited to the *Nfkbia* promoter in the absence of Trap-80, but at a reduced level, mirroring the lower level of p65 binding noted earlier.

We also examined the recruitment of the general transcription factor IIB (TFIIB) to promoters, in the presence and absence of Trap-80. TFIIB is an essential component of the pre-initiation complex, shown in vitro to be required for the recruitment of pol-II [20]. Consistent with this, TFIIB appeared at the *Ip-10* and *Mip-2* promoters concomitantly with pol-II in wild-type fibroblasts (Figure 3C), although at later time points the relative levels of promoter-associated TFIIB declined. In Trap-80–deficient cells, the recruitment of TFIIB to the *Mip-2* promoter was unimpaired (and even slightly augmented; Figure 3C). However, TFIIB levels at the Trap-80–dependent *Ip-10* promoter were severely reduced, foretelling the failure of this promoter to recruit pol-II.

Since Trap-80 seemed not to be required for the recruitment of pol-II or TFIIB to Trap-80–independent promoters, we wondered whether a Mediator complex containing Trap-80 associates with these promoters at all. To check this, we used antibodies against Trap-80 to examine its presence at promoters by ChIP. As expected, we detected Trap-80 at the promoters for the Trap-80–dependent *Ip-10* and *Il-6* genes (Figure 3D). We also found Trap-80, though, at the *Mip-2* and *Nfkbia* promoters, despite the fact that these genes can still be expressed normally in cells where Trap-80 levels have been knocked-down. This suggests that a Mediator complex which ordinarily contains Trap-80 is involved in transcriptional activation at all of these promoters, but that the Trap-80 subunit is functionally essential only at some of them. However, one caveat to this interpretation is that the apparent difference in Trap-80 dependency between the two classes of promoters might be only quantitative, and the seemingly Trap-80–independent *Mip-2* and *Nfkbia* promoters might actually manage to bind to the low level of residual Trap-80 remaining in the knock-down cells. To deal with this concern, we also checked for the presence of Trap-80 at these promoters in Trap-80–deficient cells. Trap-80 was undetectable at any promoters in Trap-80 knock-down cells, including those of *Mip-2* and *Nfkbia*—confirming that it is truly dispensable for the expression of these genes.

In Trap-80–deficient cells, the Mediator complex is “invisible” when using antibodies against Trap-80. We therefore used antibodies against another component, Med-26, to assess the involvement of the Mediator complex when Trap-80 is missing. After stimulation of Trap-80–deficient cells we could still find roughly normal levels of Med-26 at the *Mip-2* promoter, confirming that its transcription involves the Trap-80–independent participation of Mediator, and also serving as a control that in these cells the Mediator complex is not drastically disrupted (Figure 3E). In these cells, however, Med-26 was undetectable at the Trap-80–dependent *Ip-10* promoter. This supports the notion that the inability of p65 to interact with the Mediator complex in Trap-80–deficient cells underlies their failure to transcribe Trap-80–dependent genes.

Interestingly, we noticed that Trap-80 was present on promoters at above-background levels in resting wild-type cells, preceding the stimulus-induced promoter-binding by NF-κB (Figure 3D; compare with Figure 3A). This was particularly apparent at the promoters for *Nfkbia* and *Ip-10*, and in parallel experiments in which HA-Trap-80 was stably over-expressed by around 10–100×, we could also detect HA-Trap-80 at the *Mip-2* and *Il-6* promoters before stimulation (Figure S8). In contrast, we detected Med-26 at the *Ip-10* and *Mip-2* promoters only after transcription was induced by stimulating wild-type cells with TNF-α (Figure 3E). The Med-26 subunit is associated with an active subcomplex of Mediator that is able to bind pol-II, and which accounts for its transcriptional cofactor activity in vitro [21–24]. Our results indicate that while some Mediator seems to be preloaded on promoters in vivo, as has recently been described in yeast [25], contact with p65 is required for the establishment of an active, Med-26-containing complex at target promoters upon stimulation.

Taken together, our data indicate that one mechanism of transcriptional activation by p65 depends on its direct interaction with Mediator, and that this is essential for expression of a subset of its target genes in vivo. Without Trap-80, p65 binding to the promoters of these genes is not prevented, but once bound it is unable to interact with the Mediator complex, and thereby drive the recruitment of pol-II and the initiation of transcription.

### Artificial Contact with Mediator Can Bypass the Requirement for Trap-80

Three predictions arise from this model: first, the binding sites for p65 should be situated close to the transcriptional start sites of Trap-80–dependent promoters. We analysed the TNF-α–induced genes revealed by the microarray, and could identify conserved (between mouse and human) NF-κB binding motifs with high confidence in 85% of Trap-80–dependent promoters. At >92% of these, the promoter-proximal site lies within 800 bp of the transcriptional start site (see later), consistent with a direct role for p65 in interacting with Mediator to recruit pol-II.

**Figure 4:**
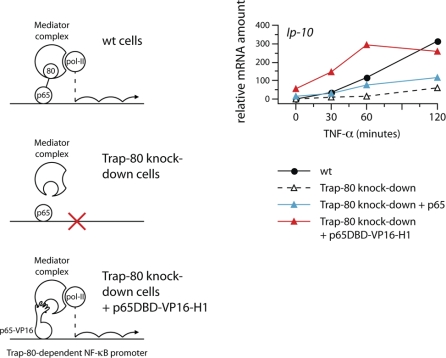
Artificial Contact with Mediator Rescues Expression of Trap-80–Dependent Genes The experimental rationale is depicted schematically on the left. On the right, Trap-80 knock-down fibroblasts were transduced with a retrovirus driving expression of full-length p65 (blue triangles), or of a fusion of the p65 DBD with the H1 region of VP16 (p65DBD-VP16-H1; red triangles), and expression of *Ip-10* mRNA was compared to wild-type (wt) and untransduced Trap-80 knock-down cells. The result is representative of five experiments. Similar fusions using both the H1 and H2 regions of VP16, or fusions to full-length p65, gave the same results (not shown).

Second, one should be able to bypass the need for Trap-80 by artificially recruiting an alternative transcriptional activation domain capable of interacting with a different Mediator subunit, to Trap-80–dependent promoters (schematically depicted in Figure 4). To attempt this, we chose to use the well-studied transcriptional activation domain of the herpes simplex virus VP16 protein. Transcriptional activation by the VP16 TAD depends on its direct interaction with the Med25 subunit of the Mediator complex, which can occur through either of two subregions (H1 and H2 [26]). Also, the only proteins it can pull-down from total nuclear extracts are Mediator components [10], implying that additional, unwanted interactions with other nuclear constituents are weak or nonexistent. To effect recruitment to NF-κB target promoters, we used the Rel homology region of p65 (p65 DBD, encompassing both its DNA-binding domain and also the region required for regulation by IκBα). When over-expressed in wild-type cells, the p65 DBD is able to out-compete full-length p65 for binding to κB sites in promoters, and acts as a dominant-negative allele (Figure S9). We generated retroviruses encoding fusion proteins between the p65 DBD and the H1 region of the VP16 TAD, since the H2 region has been shown to make nonessential contacts with Trap-80 [4,26]. After stimulation with TNF-α, Trap-80–deficient fibroblasts transduced with a control virus encoding full-length p65 still showed severely impaired *Ip-10* expression compared to wild-type fibroblasts (although the over-expression of p65 did slightly increase *Ip-10* levels above those seen in untransduced cells; Figure 4). Expression of the p65 DBD fused to the H1 region of VP16, however, fully restored *Ip-10* expression in the absence of Trap-80, to levels that even exceeded those seen in wild-type fibroblasts (Figure 4). Thus, when contact between p65 and Mediator is prevented by the absence of Trap-80, artificial contact with a different Mediator subunit is sufficient to rescue expression of a Trap-80–dependent NF-κB target gene.

### Mutations of p65 That Prevent Interaction with Trap-80

**Figure 5:**
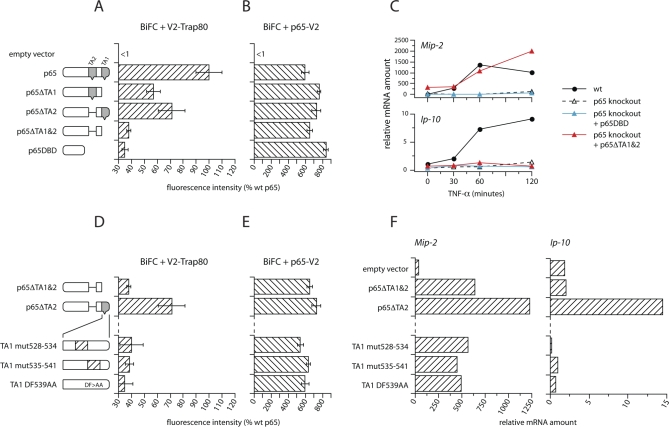
Mutations of p65 That Prevent Interaction with Trap-80 (A) Deletion of TA1 and TA2 prevents the interaction of p65 with Trap-80. (B) All p65 deletion mutants tested can still homodimerize. (A, B, D, and E) depict fluorescence intensities of HEK-293 cells after co-transfection with vectors expressing the indicated mutants of p65 fused to Venus fragment 1 (V1), and either V2-Trap-80 (A and D) or p65-V2 (B and E). Intensities are expressed as a percentage of the level in cells co-expressing p65-V1 and V2-Trap-80 (shown in [A]). The residual ∼30% fluorescence seen with the p65 DBD in (A) is similar to the level seen in cells co-expressing V1 and V2 fused to control, noninteracting proteins. Error bars indicate standard errors of independent transfections. The results presented here are representative of two to four experiments. (C) Deletion of TA1 and TA2 prevents expression of *Ip-10* but does not affect expression of *Mip-2*. p65-knockout fibroblasts were transduced with retroviruses driving expression of the p65 DBD (blue triangles) or of p65ΔTA1&2 (red triangles), and expression of *Ip-10* and *Mip-2* was compared to wild-type and untransfected p65-knockout cells. (D) Mutations in TA1 that prevent the interaction of p65ΔTA2 with Trap-80. (E) All p65 mutants tested can still homodimerize. (F) Mutants that do not interact with Trap-80 cannot drive expression of *Ip-10*, but expression of *Mip-2* is unaffected. Expression of *Mip-2* (left) and *Ip-10* (right) mRNA in p65-knockout fibroblasts transduced with retroviruses expressing the indicated p65 mutants, after stimulation for 1 h with TNF-α.

The third prediction is that it should be possible to mimic the absence of Trap-80 at NF-κB–dependent promoters by introducing mutations into p65 that disrupt its interaction with Trap-80. Two transcriptional activation regions have previously been identified within the carboxy-terminus of p65 (TA1 and TA2 [8,9]). We generated mutant forms of p65 in which either or both of these regions were deleted, and assayed their in vivo interaction with Trap-80 using BiFC. As a negative control we used the p65 DBD, which lacks the entire carboxy-terminus. All mutants were expressed at comparable levels, as detected by western blotting (unpublished data), and interacted to similar extents with full length p65 (Figure 5B). However, deletion of either TA1 or TA2 alone each diminished the interaction with Trap-80, and deletion of both together (p65ΔTA1&2) completely reduced it to background levels (Figure 5A). We next tested the ability of each mutant to rescue NF-κB target gene expression in TNF-α–stimulated p65-knockout fibroblasts. Transduction with viruses encoding full-length p65, or p65 with deletions of either TA1 or TA2 alone, restored transcription of both the *Ip-10* and *Mip-2* genes to levels that equalled or even exceeded those in wild-type cells (Figure S10). Notably though, the p65 mutant lacking both TA1 and TA2 was completely unable to drive transcription of the Trap-80–dependent *Ip-10* gene, but it could still activate expression of the Trap-80–independent *Mip-2* gene to wild-type levels (Figure 5C). Thus, p65 can activate transcription of Trap-80–dependent and –independent genes using separable regions within its carboxy-terminus. These findings can be explained by an inability of the p65ΔTA1&2 mutant to interact with Mediator. However, since it could also be argued that deletion of a substantial domain from p65 may have other, additional consequences for the protein's function, we sought to identify more subtle mutations in which interaction with Trap-80 was still disrupted. We used the p65 mutant lacking TA2 as a template, since this protein drives transcription of *Ip-10* and *Mip-2* normally, but depends on TA1 for its interaction with Trap-80 (Figures 5A and S10). By initially substituting blocks of seven amino acids within TA1 (e.g., TA1 mut528–534 and TA1 mut535–541), and subsequently by mutating adjacent pairs of amino acids, we were able to identify a p65 mutant in which only two amino acid changes result in the abolition of the interaction with Trap-80 (TA1 DF539AA; Figure 5D). This mutant can still activate transcription of the Trap-80–independent *Mip-2* gene, but is inactive at the Trap-80–dependent *Ip-10* promoter (Figure 5F).

Thus, using two independent approaches—knock-down of Trap-80 and targeted mutation of the p65 carboxy-terminus—we find that contact with the Mediator complex through the Trap-80 subunit is responsible for transcriptional activation by p65 at a subset of its target genes in vivo.

### p65 Controls the Recruitment of Secondary Transcription Factors

**Figure 6:**
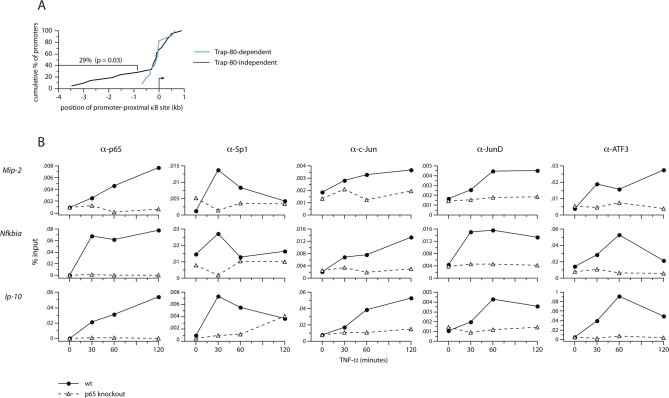
p65 Is Required for Promoter Occupancy by Secondary Transcription Factors (A) Positions of promoter-proximal NF-κB binding motifs relative to the transcriptional start site. Cumulative percentage of promoters in which the closest conserved NF-κB binding motif lies within the indicated distance to the TSS. Promoters analysed are those of the top 30 Trap-80–dependent and 30 Trap-80–independent genes, from amongst the 200 most induced by TNF-α. (B) Recruitment of p65 and secondary transcription factors to the *Mip-2*, *Nfkbia,* and *Ip-10* promoters in wild-type and p65-knockout fibroblasts. ChIP using antibodies against p65, Sp1, c-Jun, JunD, or ATF-3, after stimulation with TNF-α. The results presented here are representative of two to three experiments.

The observation that expression of many endogenous target genes (including *Mip-2* and *Nfkbia*) is unimpaired in Trap-80–deficient fibroblasts, though, indicates that p65 can utilize a second mode of transcriptional activation at these promoters, which does not depend on either of the transcriptional activation domains identified in earlier in vitro studies. To try to uncover features that might explain their different requirements for activation by p65, we compared the promoters of Trap-80–dependent and –independent TNF-α–induced genes identified by our microarray analysis. We could identify conserved κB motifs in a similar fraction of Trap-80–dependent and –independent TNF-α–induced genes (85% versus 92%, respectively), and the consensus sequence did not obviously differ between the two (Figure S11). However, for a substantial fraction of Trap-80–independent promoters, the most proximal predicted κB site was >1 kb from the transcriptional start site (29%; Figure 6A). This is significantly different from the Trap-80–dependent genes, and suggests p65 may not be directly involved in assembly of the pre-initiation complex at these promoters. With this in mind, we investigated whether the two classes of promoters could be distinguished by the presence or absence of binding motifs for other transcription factors. Although we were unable to find any clear-cut motifs that could unambiguously discriminate between Trap-80–dependent and –independent promoters, there were clear differences in the “signatures” of transcription factor binding sites associated with the two promoter classes (Figure S12 and Table S2). Trap-80–independent promoters were highly enriched for the presence of GC-box motifs (the binding site for Sp1 and related transcription factors) compared with total mouse promoters, although this enrichment did not reach statistical significance when compared with Trap-80–dependent promoters. On the other hand, Trap-80–dependent promoters were themselves strongly enriched for the presence of a TATA-box, and for binding sites for the Ap-1 and HSF families of transcription factors. All promoters induced by TNF-α contained statistically elevated levels of NF-κB-binding and E-box motifs when compared with total mouse promoters.

These findings prompted us to investigate the co-occupancy of endogenous promoters by other transcription factors alongside p65. The promoters for *Mip-2* and *Nfkbia*, as well as that of *Ip-10*, contain putative binding sites for Ap-1, ATF/CREB, and Sp1, in addition to NF-κB. We performed ChIP using antibodies against c-Jun and Jun-D (which form part of Ap-1), ATF-3, and Sp1. All of these transcription factors were recruited to both the Trap-80–independent *Mip-2* and *Nfkbia* promoters, and the Trap-80–dependent *Ip-10* promoter, upon stimulation of fibroblasts with TNF-α (Figure 6B). Remarkably, in every case, binding to these promoters was totally abolished in p65-knockout fibroblasts. This effect was specific for NF-κB–dependent genes, since Sp1 remained bound to the promoters of control, housekeeping genes in both wild-type and p65-knockout cells (Figure S13 [27]). Thus, at native NF-κB target promoters, the initial recruitment of p65 is required for the subsequent binding of other, secondary transcription factors.

To explore whether this phenomenon also occurs in another cell type, we used lipopolysaccharide (LPS)-stimulated primary dendritic cells (DCs), derived in vitro using cells from wild-type and p65-knockout mice. Unlike the situation in fibroblasts, many NF-κB target genes are expressed in DCs in the absence of p65; however, the *Vcam-1* and *Ip-10* genes are still largely p65-dependent (Figure S14A). The promoters for both of these genes contain binding sites for Ap-1, and in wild-type DCs both are able to recruit c-Jun upon LPS stimulation (Figure S14B). As we had observed in fibroblasts, though, binding to both promoters was prevented in p65-knockout DCs.

### An Artificially Recruited Secondary Transcription Factor Drives Transcription in the Absence of p65

**Figure 7:**
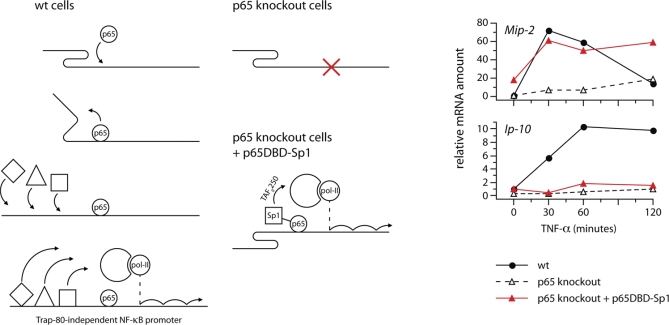
An Artificially Recruited Secondary Transcription Factor Drives Transcription in the Absence of p65 On the left is a cartoon illustrating our model for the events occurring at Trap-80–independent promoters. In wild-type cells, initial p65 binding brings about an undefined change to target promoters, which allows the binding of various secondary transcription factors. Once bound, these can drive the recruitment of pol-II, and so enable transcription. In p65-knockout cells, secondary transcription factors cannot bind and no transcription takes place. If binding of a secondary transcription factor is artificially restored (in this case Sp1), transcription is rescued at promoters that can recruit the relevant co-factors (in this case TAF_II_250). On the right, p65-knockout cells were transduced with a retrovirus expressing a fusion of the p65 DBD with the Sp1 TAD (red triangles, p65DBD-Sp1), and expression of *Mip-2* and *Ip-10* mRNA was compared to untransduced wild-type and p65-knockout fibroblasts. The result is representative of three experiments.

The above results indicate that one mechanism by which p65 could drive transcriptional activation at Trap-80–independent promoters would be by controlling the recruitment of secondary transcription factors whose activities do not require Trap-80 (as illustrated in Figure 7). If this explanation is correct, we should be able to rescue expression of Trap-80–independent genes in p65-knockout fibroblasts by bringing one of the relevant transcription factors to their promoters. We decided to attempt this using the transcriptional activation domain from Sp1. Sp1 is recruited in a p65-dependent fashion to NF-κB target promoters (Figure 6B). The binding site for Sp1 is frequently found in Trap-80–independent promoters (GC-box; Figure S12), and it has been implicated in the expression of several NF-κB–regulated genes (e.g., *Mcp-1* [28]). Moreover, while the Sp1 TAD requires Mediator for its activity [29], it does not directly interact with the Mediator complex, so we reasoned that it was unlikely to show a particular dependency on the Trap-80 subunit.

Using a similar strategy to that used earlier (Figure 4), we generated retroviruses encoding a fusion protein between the p65 DBD and the Sp1 TAD, and used these to infect p65-knockout fibroblasts. Expression of the Trap-80–independent *Mip-2* gene was completely restored to wild-type levels in infected cells (Figure 7). This demonstrates that recruitment of Sp1, an event that is normally controlled by p65, is sufficient to drive transcription even in an experimental setting in which p65 itself is absent. Therefore, the ability of p65 to control the binding of secondary transcription factors such as Sp1 to target gene promoters constitutes a second, indirect, mode of transcriptional activation, independent from its direct interaction with Mediator via Trap-80.

Although Sp1 binding sites are enriched at the promoters of Trap-80–independent genes, there also exist instances at those of Trap-80–dependent genes (e.g., *Ip-10*, Figure 6B). In such cases, binding of Sp1 to the promoter (along with other transcription factors) is not sufficient to drive transcription in Trap-80–deficient cells. In line with this, artificial recruitment of the Sp1 TAD to the Trap-80–dependent *Ip-10* promoter failed to restore its expression in p65-knockout cells (Figures 7 and S15). A difference between the Trap-80–independent and Trap-80–dependent genes, then, corresponds to the ability of secondary transcription factors (exemplified here by Sp1) to drive their transcription following p65-dependent recruitment.

This raises the question of why promoter-bound Sp1 cannot drive transcription of Trap-80–dependent genes, such as *Ip-10*. Transcriptional activation by Sp1 in vitro depends on its direct interaction, through TAF_II_110, with a TFIID complex containing TAF_II_250 [30]. However, not all transcriptionally active genes in human cells are found in association with TAF_II_250, nor in yeast cells with its homologue TAF_II_145 [31–33]. We therefore examined TAF_II_250 occupancy at the *Ip-10* and *Mip-2* promoters. We found that TAF_II_250 is recruited to the endogenous *Mip-2* promoter upon stimulation with TNF-α, but no such recruitment was seen at the promoter for *Ip-10* (Figure S16). Thus, the differential responsiveness of these two promoters to bound Sp1 can be explained by their respective abilities to recruit a TFIID complex containing TAF_II_250; this, in turn, accounts for the ability of p65 to activate transcription of *Mip-2*, but not *Ip-10*, in the absence of Trap-80. Differential TAF usage by promoters may represent a widely used additional level of control over the activity of bound transcription factors. It has been shown in both yeast and mammals that promoters differ in their requirement for a TFIID complex containing TAF_II_250/145 [15,34,35]. Although the correlation is not absolute, one predictive factor for TAF-independence is the presence of a TATA-box, and it is worth noting that this motif is enriched in Trap-80–dependent promoters (Figure S12 and Table S2). However, just as there does not appear to be any single transcription factor binding motif that unequivocally separates the two classes of promoter, the association of Trap-80–independent promoters with TAF_II_250 presence is not perfect, and there exist some Trap-80–dependent genes whose human counterparts are bound by TAF_II_250 (e.g., *Adm* and *Cebpb* [31]), and some Trap-80–independent promoters which contain a TATA-box (e.g., *Ccl7*). Thus, while Sp1 serves as a successful example in the case of the *Mip-2* promoter, we certainly do not suggest that *all* Trap-80–independent transcription is mediated by the same secondary transcription factor.

Rather, our data indicate that each NF-κB–dependent promoter contains a combination of sites for the binding of various transcription factors, any of which could drive transcription if present and active in that promoter context. Importantly, though, in fibroblasts and DCs this binding is subject to overall upstream control by p65, and it is likely that this reflects a general mechanism used by NF-κB–dependent promoters in other cell types.

## Discussion

When the studies described here were initiated, numerous in vitro data were known about transcription by NF-κB, but the actual mechanism of transcription downstream of p65 binding to endogenous genes in vivo was unclear. Since the Mediator complex was known to play an important role in most, if not all, transcription by pol-II, we set out to disrupt its interaction with p65, as a means to dissect p65-driven transcription. We found that expression of some NF-κB target genes depends on direct contact between p65 and Mediator, which occurs through the Trap-80 subunit and the TA1 and TA2 regions of p65. This contact is needed for the establishment of an active, Med-26–containing Mediator complex at promoters, recruitment of TFIIB and pol-II, and thereby the initiation of transcription. While this result is not surprising, it does provide important confirmation that events hitherto only described in minimalist in vitro experiments are necessary, and sufficient, for the expression of some genes in their natural context in vivo.

The finding that 3T3 fibroblasts remain viable even after depletion of Trap-80 to <10% of normal levels is remarkable, considering that its yeast homologue Srb4 is required for most, if not all, transcription by pol-II [15]. Srb4 is essential for the integrity of the yeast Mediator complex, which, without it, dissociates at the boundary between the structurally conserved head and middle modules [36,37]. In mammalian cells, like in yeast, the Mediator complex is critical for pol-II–driven transcription: its addition is required for the in vitro activity of various transcriptional activators [10,29], and its depletion from mammalian nuclear extracts abolishes all transcription by pol-II [7,38]. Hence, the survival of fibroblasts after Trap-80 has been knocked-down implies that some Mediator activity must still remain. One possibility is that the amount of cellular Mediator is not normally limiting for the expression of essential genes, and that the residual 5%–10% level of Trap-80 in knock-down cells suffices for these. However, we find that the expression of >96% of transcripts changes by less than 1.5-fold in Trap-80–deficient cells (Figure 2B). This finding argues that, instead, Trap-80–deficient cells contain Trap-80–less, but otherwise functional, Mediator complexes. Proteomic analyses have so far identified Trap-80 to be part of a common core of subunits, shared by all Mediator species [23,39]. It seems likely, then, that Trap-80 does not play the same essential structural role as yeast Srb4, and that Mediator complexes that normally incorporate Trap-80 are still able to at least partially assemble when this subunit is missing. This interpretation is supported by our finding that the Mediator complex, revealed by the Med-26 subunit, is recruited to the *Mip-2* promoter even in Trap-80–deficient cells (Figure 3E).

We have shown that the interaction of p65 with Mediator through Trap-80 is sufficient to drive transcription. However, the discovery that a subset of p65-dependent genes are transcribed normally even when the interaction of p65 with Mediator is abolished was completely unanticipated. Moreover, a mutant form of p65 that not only cannot interact with Trap-80, but that also lacks both previously identified transcriptional activation domains, can still activate the Trap-80–independent *Mip-2* gene in vivo (p65ΔTA1&2, Figure 5C). This finding prompted us to examine more closely the events that occur at promoters upon engagement of NF-κB. Remarkably, we found that without the binding of p65, NF-κB target promoters cannot be bound by many other transcription factors. Thus, it appears that a p65-containing NF-κB dimer binds to target promoters as a lone, “pioneer” transcription factor, and controls their subsequent co-occupancy by secondary transcription factors (illustrated in Figure 7).

One model for this, which we do not favour, could be that secondary transcription factors bind to promoters via direct, co-operative interactions with p65. Such a scenario has been previously shown in the context of particular promoters containing juxtaposed binding sites (e.g., HIV1-LTR [40], *Ifnb1* [41]), but this arrangement is not a general feature of NF-κB target promoters. Moreover, it seems unlikely that pairwise interactions with p65 could account for the binding of multiple transcription factors to each of many different promoters (and at non-NF-κB target promoters, co-operative binding with p65 is clearly not required; see Figure S13).

A more plausible possibility is that p65 controls promoter accessibility by inducing local alterations to chromatin. In macrophages, NF-κB–driven activation is accompanied by nucleosome remodeling at target gene promoters [42]. However, we could detect no differences in promoter accessibility to micrococcal nuclease digestion after stimulation of wild-type and p65-knockout fibroblasts (unpublished data). Alternatively, p65 binding may bring about changes to histone modifications, several of which have been described to be associated with the expression of NF-κB target genes in different systems (e.g., lysine acetylation [43,44] and methylation [45], serine phosphorylation [46,47]). Further experiments are required to determine whether these could account for the control over secondary transcription-factor binding.

In p65-knockout cells, artificial recruitment of a secondary transcription factor is sufficient to restore gene expression (Figure 7), indicating that regardless of the mechanism, the regulation of promoter occupancy constitutes a second, independent mode of transcriptional activation by p65.

What, though, could be the benefit of having a second mode of transcriptional activation? After all, in real, nonexperimentally manipulated cells, an intact Mediator complex containing Trap-80 is always present. We can envisage two situations in which the ability of p65 to control recruitment of secondary transcription factors to a promoter could be important. First, if the only means by which p65 could activate transcription was through its direct binding to Mediator, then the transcriptional output at every NF-κB−dependent promoter should be the same, and upon its release from promoters, transcription would necessarily halt. There are numerous mechanisms that control the longevity of promoter-bound p65, including nuclear export by resynthesized IκB molecules [48], ubiquitination and proteasomal degradation [49], and replacement by other NF-κB dimer species [50]. Considering the tremendous diversity of NF-κB target genes, though, it seems inconceivable that the optimal biological window and level of expression for all of them can be identical (and experimentally this is not the case; compare, for example, expression of *Mip-2* and *Il-6* in Figure 2A). By endowing p65 with the ability to license promoters for the binding of secondary transcription factors, there is a means to customize expression levels, and prolong transcription after the departure of p65. From the point of view of a promoter, this would be an attractive solution, since NF-κB–dependence can be retained at the same time as tailoring the expression pattern by selecting binding sites for appropriate secondary transcription factors.

Second, κB sites are not always located in promoters close to the transcriptional start sites, and in some cases can be several kilobases away (Figure 6A; examples include *Mcp-1* and *JunB*). At such a distance, looping of the intervening DNA would be required to bring bound p65 into the proximity of core promoter elements, and this may not allow a sufficiently stable interaction with Mediator to enable nucleation of the pre-initiation complex. However, the local presence of p65 is nevertheless adequate to regulate the recruitment of secondary transcription factors. In turn, these newly arrived transcription factors can stably bind to the promoter, and themselves interact with components of the pre-initiation complex to drive transcription. In this model, the Trap-80–independent mode of activation by p65 is critical to permit it to operate at enhancers.

A corollary of activation by p65 in this way is that the activity of a given target promoter, although entirely NF-κB–dependent, will depend on the availability of suitable secondary transcription factors. Since this is determined by both cell-type and stimulus, this mode of activation is likely to be essential to allow genes controlled by NF-κB to attain an appropriate pattern of expression in different biological contexts.

## Materials and Methods

### Plasmids.

For expression in yeast, fragments from the N- or C termini of p65 (NT: amino acids [aa] 1–305; CT1: aa 306–549, CT2: 431–549) were cloned into pAct2. Full-length Trap-80, and amino or carboxy terminal fragments (NT: aa 1–335, CT: aa 336–649) were cloned into pGBT9. For expression in HEK-293 cells, the coding sequences for full-length mouse p65 and Trap-80 were cloned in pCDNA3. Trap-80 was tagged at the N terminus with the HA epitope MYPYDVPDYA. For BiFC, p65 and mutants thereof were fused to Venus fragment 1 (V1: aa 1–158) or fragment 2 (V2: aa 159–239) using the linker sequence SRGSGGGGSGGGGSSG, and Trap-80 was fused to V2. p65 mutations are as follows: ΔTA1 is truncated at aa 519; ΔTA2 lacks aa 441–474; mut528–534 and mut535–541 each substitute 7 aa for AAASAAA; DF539AA substitutes aa 539 and 540 for AA (numbers refer to aa positions in full-length p65). Trap-80 was knocked-down using hairpins directed against the sequences AGAGATGGTCGGGTAATCA or GACATTGGTGATCTTGGCA (in the Trap-80 CDS), cloned into pSuper-Retro-Puro. The shRNA-resistant Trap-80 contains two silent point mutations (underlined): AGAGACGGTCGGGTCATCA, and was cloned in pMY-IRES-GFP. For generation of biotin-tagged Trap-95, the Escherichia coli BirA coding sequence was cloned into pMY-IRES-Bsd (conferring resistance to blasticidin), and full-length Trap-95 was tagged at the C terminus with the peptide GLNDIFEAQKIEWH, and cloned in pMY-IRES-Tomato (expressing red fluorescent Tomato protein). For expression in fibroblasts, HA-Trap-80, full-length p65 and mutants thereof, and the p65 DBD (aa 1–305), either alone or fused to VP16-H1 (aa 411–456) or Sp1 TAD (aa 92–551), were all cloned in pMY-IRES-GFP.

### Antibodies.

Polyclonal antibodies against HA, p65, pol-II Rbp1 subunit, Trap-80, Med-26, TFIIB, ATF3, c-Jun, Jun-D, Sp1, and c-Rel were from Santa Cruz, that against TAF_II_250 was from Abcam. Monoclonal anti-p65 (N terminus) was from Santa Cruz. Monoclonal anti-HA is produced by the hybridoma 12CA5.

### Yeast 2 hybrid.

Y153 yeast were grown at 30 °C in YAPD medium and transformed using lithium acetate. Transformants were selected by growth on YNB plates without tryptophan or leucine, and additionally lacking histidine and containing 25 mM 3-amino triazole to select for interactions between hybrid proteins. Expression of LacZ was screened by transfer of colonies to nitrocellulose, lysis, and incubation at 37 °C with X-Gal.

### Cell culture.

HEK-293 and Ecotropic-Phoenix cells were were transfected using CaPO_4_. BiFC fluorescence intensities in transfected cells were measured by flow cytometry. 3T3 cells were infected with retroviral supernatants from Ecotropic-Phoenix packaging cells. Retroviral gene expression was monitored using flow cytometry to measure co-expressed fluorescent proteins. Where necessary, cells were sorted to obtain equivalent expression levels. Primary DCs were generated from foetal liver progenitor cells by culture for 8–10 d with GM-CSF (4% supernatant from transfected X63 cells). Cells were stimulated with 5 ng ml^−1^ mouse TNF-α or with 100 ng ml^−1^ LPS.

### Immunoprecipitations and pull-downs.

Nuclei were isolated by cell lysis in L1 buffer (50 mM Tris, 2 mM EDTA, 0.1% NP-40, 10% Glycerol [pH8]) and nuclear proteins were extracted using L1 +250 mM NaCl for 10 min. After centrifugation, the salt in the supernatant was diluted to 100 mM. For immunoprecipitations, extracts were incubated overnight with 2 μg antibody followed by 30 min with 5 μl protein A- or protein G-sepharose, per 100 μg total protein. For pull-down of in vivo biotinylated Trap-95, extracts were incubated with 2 μl streptavidin-M280 magnetic beads (Dynal) per 100 μg total protein. Bound material was washed in L1 +150 mM KCl and analysed by western blotting.

### Microarray analysis.

Total RNA was prepared with RNeasy (Qiagen) from three independent samples per group, and used to prepare labelled ss-cDNA for hybridization on Affymetrix Mouse Gene 1.0 ST microarrays. The data have been deposited at the National Center for Biotechnology Information (NCBI) Gene Expression Omnibus (http://www.ncbi.nlm.nih.gov/geo), with accession number GSE12697. Only transcripts whose microarray Δ log signals were reproducible between groups for all samples (σ/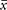 < 0.5 and *p* < 0.1) were considered for analysis. A set of 36 transcripts were measured by RT-PCR from the same samples, and used to generate a standard curve (*r =* 0.84) to quantify the microarray probe signals. NF-κB binding motifs conserved between mouse and human were identified in the region −9,000 to +1,000 bp relative to the mouse transcriptional start site (TSS) using Consite (matrices MA0061, 0101, and 0107, conservation >70%, TF score >85% [51]). The fraction of promoters for which the most proximal κB site was >1 kb (kilobase pair) from the TSS were compared using the Fisher's exact test. Over-represented motifs in sets of promoters were identified using MotifSampler [52].

### ChIP and PCR.

ChIP was performed as described [53], using primers which amplify promoter regions within 300 bp upstream of the TSS (binding sites for the transcription factors studied all lie within ±500 bp of the TSS at the promoters analysed). All PCR was performed using quantitative real-time analysis with gene-specific fluorescent probes. Primer sequences are available on request.

## Supporting Information

Figure S1 p65 Interacts with Trap-80 (A) Yeast two-hybrid experiments. Yeast cells were sequentially transformed with plasmids encoding the Gal4 activation domain fused to either the amino terminus of p65 (p65 NT) or two nested carboxy terminal fragments (p65CT1/CT2), followed by the Gal4 DNA-binding domain fused to either full length, or amino- or carboxy-terminal Trap-80 (Trap-80 NT and Trap-80 CT). The blue colour of colony lifts when Trap-80 is combined with p65 CT2, or when Trap-80 NT is combined with p65 CT1 or CT2 is indicative of an in vivo interaction between the two hybrid proteins. The failure of p65 CT1 to drive Lac*Z* expression when co-transformed with full-length Trap-80 is probably due to its weak expression: western blotting of yeast cell extracts using antibodies specific for the carboxy-terminus of p65 revealed only very low amounts of the shorter p65 fragment (CT2), and undetectable levels of the longer fragment (CT1, not shown). Yeast co-transformants containing all combinations of full-length Trap-80 or Trap-80 NT, combined with p65 CT1 or CT2, grew on medium lacking histidine (not shown).
(B) Co-immunoprecipitation of p65 and Trap-80. HEK-293 cells were co-transfected with expression vectors for p65 and a haemagglutinin (HA) epitope-tagged allele of Trap-80, and nuclear extracts were prepared after stimulation with TNF-α to induce nuclear entry of NF-κB. p65 was detected by western blotting after immunoprecipitation using an anti-HA antibody, or without antibody (left panel). In the reciprocal experiment, tagged Trap-80 was detected with anti-HA after immunoprecipitation using anti-p65 (right panel). The level of p65 in total nuclear extract is shown as “input”; HA-Trap-80 was not easily detectable in nuclear extracts before immunoprecipitation.
(C) BiFC mediated by in vivo interaction between p65 and Trap-80. HEK-293 cells were transfected with vectors expressing Venus fragment 2 fused to the N terminus of Trap-80 (V2-Trap-80; i, iii, iv), Trap-80–V2 (v), or p65-V2 (vi), either alone or together with p65-V1 (i, ii, vi), p65DBD-V1 (iv), or V1-p65 (v). Fluorescence from either p65-V1 or V2-Trap-80 was undetectable when transfected alone (ii, iii). (iv) and (v) are controls for the specificity of the interaction based on the yeast two-hybrid results (above), wherein the interacting carboxy-terminus of p65 is deleted (iv), or the two proteins are fused at the noninteracting termini (v). The residual ≈30% fluorescence seen in these cases is similar to the level seen in cells co-expressing V1 and V2 fused to control, noninteracting proteins. The strong self-association of p65 into homodimers serves as a positive control (vi). The fluorescence intensity of transfected cells is expressed as a percentage of the level in cells co-expressing p65-V1 and V2-Trap-80 (i). Error bars indicate standard errors of independent transfections. The results presented here are representative of three experiments.
(3.97 MB TIF)

Figure S2 Trap-80 Knock-Down Fibroblasts (A) Proliferation of Trap-80–deficient fibroblasts. Parallel cultures of fibroblasts expressing either the irrelevant shRNA, or an shRNA targeting Trap-80, were grown for 1 wk and the increase in cell number was determined. Proliferation is expressed as the percentage increase compared to the control culture; error bars indicate standard errors.
(C) Morphological appearance of Trap-80–deficient fibroblasts. Phase contrast images (original magnification 100×) taken of exponentially growing cultures of wild-type or Trap-80 knock-down fibroblasts.
(2.57 MB TIF)

Figure S3 Expression Levels of Trap-95-Biotin Intensity of Tomato fluorescence, expressed via an IRES sequence from a bi-cistronic mRNA also expressing Trap-95-biotin, in transduced wild-type (top) and Trap-80 knock-down (bottom) fibroblasts. The mean fluorescence level of the population is indicated. Trap-95-biotin itself co-migrates with one of two naturally biotinylated carboxylase proteins in mammalian cells, precluding direct detection on western blots.
(717 KB TIF)

Figure S4 Controls for the Specificity of Trap-80 Knock-Down (A) Two independent hairpins targeting Trap-80 were used to generate clonal lines of 3T3 fibroblasts. A comparable number of clones was generated using each of the shRNAs targeting Trap-80, and using an irrelevant, scrambled shRNA. The degree by which *Trap-80* mRNA was knocked-down varied from one clone to another, in the range of ≈70%–95% reduction of wild-type levels (not shown). The residual levels of *Trap-80* mRNA are shown for a single clone expressing each shRNA, which were chosen for further study. mRNA levels are expressed as a percentage of the level in control cells. Trap-80 shRNA number 1 corresponds to the shRNA used in the experiments described in the main text. Error bars indicate standard errors; the results presented here are representative of more than ten experiments.
(B) Expression of *Mip-2*, *Nfkbia*, *Ip-10*, and *Il-6* mRNA in fibroblasts expressing either of the two shRNAs targeting Trap-80, after stimulation with TNF-α.
(C) Reconstitution of Trap-80 in knock-down cells. Fibroblasts expressing an shRNA targeting Trap-80 were transduced with a retrovirus driving expression of an shRNA-resistant form of Trap-80 (red triangles; total *Trap-80* mRNA levels were around 100× greater than those of wild-type cells [not shown]), and mRNA was measured as in (B).
(3.97 MB TIF)

Figure S5 Expression of Both Trap-80–Dependent and Trap-80–Independent NF-κB Target Genes Requires p65 mRNA expression of *Mip-2*, *Nfkbia*, *Ip-10*, and *Il-6*, in normal and p65-knockout fibroblasts stimulated with TNF-α. The results presented here are representative of more than ten experiments.
(1.69 MB TIF)

Figure S6 TNF-α-Induction of Trap-80–Dependent Versus Trap-80–Independent Genes Cumulative percentage of transcripts whose expression after 1 h TNF-α stimulation exceeds that in unstimulated fibroblasts by the indicated level, expressed both as the Δ log signal of the microarray probes (upper scale) and the calculated fold induction using a standard curve generated by real-time PCR of a subset of 36 genes (lower scale). The red line represents the top 100 Trap-80–dependent transcripts; the black line represents 5000 Trap-80–independent transcripts.
(1.29 MB TIF)

Figure S7 Trap-80–Dependent and –Independent Genes mRNA expression of a subset of Trap-80–dependent (A) and Trap-80–independent (B) genes, in wild-type and Trap-80 knock-down fibroblasts.
(509 KB TIF)

Figure S8 Presence of HA-Trap-80 at Promoter Regions ChIP using antibodies against the HA epitope, from fibroblasts retrovirally over-expressing HA-Trap-80, or from control untransduced cells.
(1.88 MB TIF)

Figure S9 p65 DBD Alone Acts as a Dominant Negative Allele Wild-type 3T3 fibroblasts were transduced with a retrovirus driving expression of the p65 DBD (red triangles), and expression of *Mip-2* and *Ip-10* mRNA was compared to wild-type and p65 knockout fibroblasts. The result is representative of three experiments.
(943 KB TIF)

Figure S10 Transcriptional Activity of p65 Deletion Mutants p65 knockout fibroblasts transduced with retroviruses expressing the indicated p65 mutants were stimulated with TNF-α for 1 h, and their expression of *Mip-2* (left) and *Ip-10* (right) mRNA was compared to that of wild-type cells. Note that the level of expression of p65 mutants in transduced cells exceeds that of endogenous p65 in wild-type cells by several-fold (not shown), which may account for the heightened expression of *Mip-2*. The results presented here are representative of three experiments.
(1.61 MB TIF)

Figure S11 Consensus NF-κB Binding Motifs in Trap-80–Dependent and –Independent Promoters Logos of position weight matrices of NF-κB binding motifs conserved between mouse and human in Trap-80–dependent (top; *n =* 36) and –independent (bottom; *n =* 58) TNF-α–induced genes. Promoters analysed are those of the top 30 Trap-80–dependent and 30 Trap-80–independent genes, from amongst the 200 most induced by TNF-α.
(677 KB TIF)

Figure S12 Transcription Factor Binding Sites Associated with Trap-80–Dependent and –Independent Promoters Over-represented motifs detected in promoters of TNF-α–induced genes. See Table S2 for matrices and *p*-values. No motifs were statistically over-represented in Trap-80–independent promoters compared with Trap-80–dependent promoters.
(1.08 MB TIF)

Figure S13 p65-Independent Binding of Sp1 to the Promoters of Housekeeping Genes ChIP using antibodies against Sp1 in wild-type and p65-knockout fibroblasts. Primers were chosen to amplify the promoter regions of *MeCP2* (A) or *Tk* (B), and adjacent control regions located upstream or downstream at the indicated positions. Error bars indicate the standard errors of independent immunoprecipitations (from both unstimulated and TNF-α–stimulated cells).
(1.79 MB TIF)

Figure S14 p65-Dependent Recruitment of c-Jun to Promoters in Dendritic Cells (A) p65-dependent gene transcription in dendritic cells. mRNA expression of *Vcam-1* and *Ip-10* in normal and p65-knockout DCs stimulated with LPS.
(B) ChIP using antibodies against c-Jun.
(2.51 MB TIF)

Figure S15 p65 DBD-Sp1 Binds to Promoters p65 knockout 3T3 fibroblasts were transduced with a retrovirus driving expression of the p65 DBD-Sp1, and ChIP was performed using antibodies against the p65 N terminus.
(969 KB TIF)

Figure S16 The *Mip-2*, But Not the *Ip-10* Promoter Recruits TAF_II_250 ChIP using antibodies against TAF_II_250 in wild-type fibroblasts.
(815 KB TIF)

Table S1 Top 50 Trap-80–Dependent and Trap-80–Independent TNF-α–Induced Genes (177 KB DOC)

Table S2 Over-Represented Motifs in TNF-α–induced Gene Promoters (113 KB DOC)

## Abbreviations

BiFC: bimolecular fluorescence complementation
ChIP: chromatin immunoprecipitation
DC: dendritic cell
GTF: general transcription factor
LPS: lipopolysaccharide
pol-II: RNA polymerase II
shRNA: small hairpin RNA
TAD: transcriptional activation domain
TFIIB: transcription factor IIB
TNF: tumor necrosis factor
TSS: transcriptional start site
