# Supplementary figures and images for: Two Modes of Transcriptional Activation at Native Promoters by NF-κB p65
Source: PLoS Biol. 2009 Mar 31;7(3):e1000073. doi: 10.1371/journal.pbio.1000073 (PMC2661965; doi:10.1371/journal.pbio.1000073)

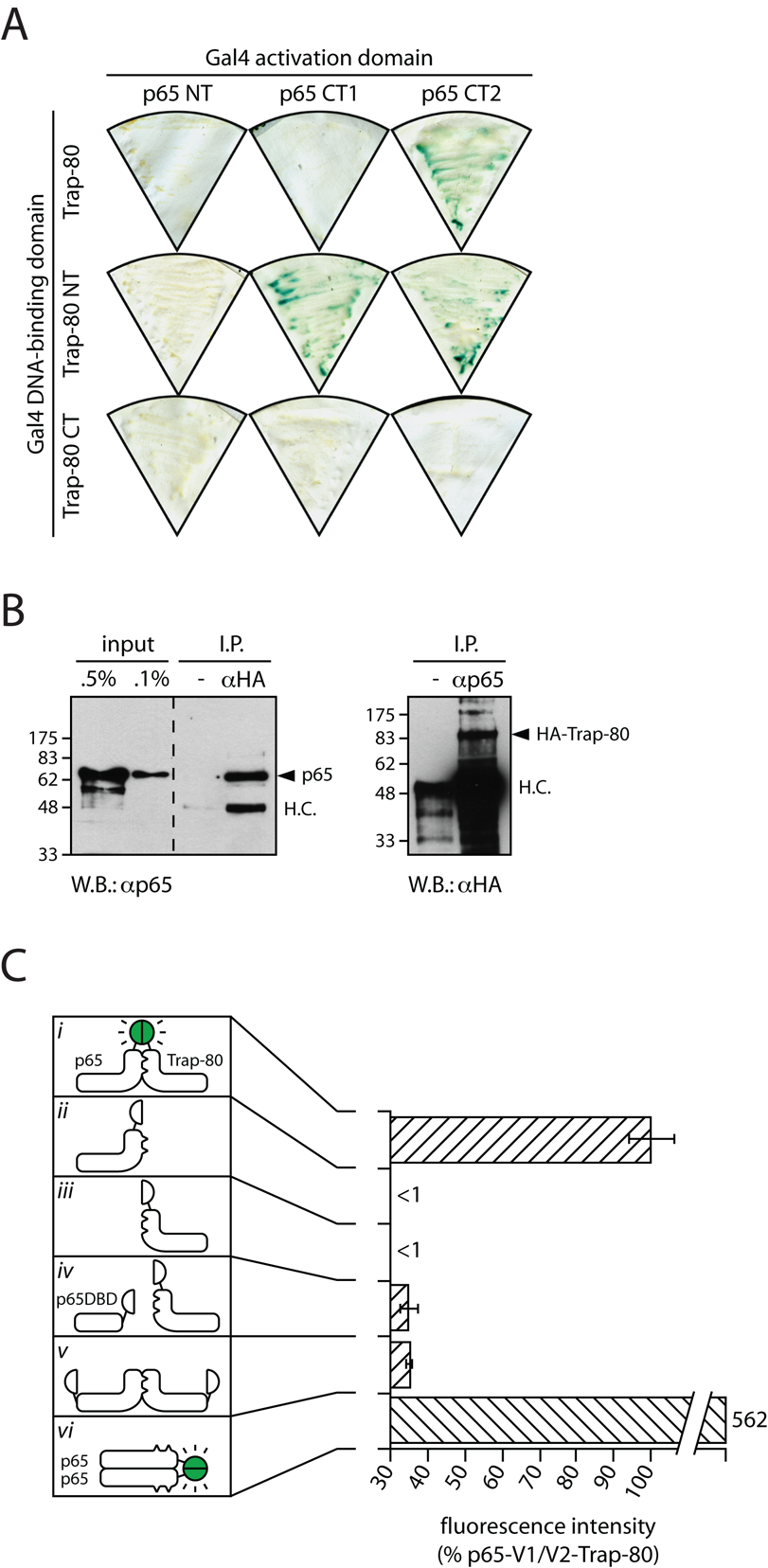

Supplement: Figure S1 — (A) Yeast two-hybrid experiments. Yeast cells were sequentially transformed with plasmids encoding the Gal4 activation domain fused to either the amino terminus of p65 (p65 NT) or two nested carboxy terminal fragments (p65CT1/CT2), followed by the Gal4 DNA-binding domain fused to either full length, or amino- or carboxy-terminal Trap-80 (Trap-80 NT and Trap-80 CT). The blue colour of colony lifts when Trap-80 is combined with p65 CT2, or when Trap-80 NT is combined with p65 CT1 or CT2 is indicative of an in vivo interaction between the two hybrid proteins. The failure of p65 CT1 to drive LacZ expression when co-transformed with full-length Trap-80 is probably due to its weak expression: western blotting of yeast cell extracts using antibodies specific for the carboxy-terminus of p65 revealed only very low amounts of the shorter p65 fragment (CT2), and undetectable levels of the longer fragment (CT1, not shown). Yeast co-transformants containing all combinations of full-length Trap-80 or Trap-80 NT, combined with p65 CT1 or CT2, grew on medium lacking histidine (not shown). (B) Co-immunoprecipitation of p65 and Trap-80. HEK-293 cells were co-transfected with expression vectors for p65 and a haemagglutinin (HA) epitope-tagged allele of Trap-80, and nuclear extracts were prepared after stimulation with TNF-α to induce nuclear entry of NF-κB. p65 was detected by western blotting after immunoprecipitation using an anti-HA antibody, or without antibody (left panel). In the reciprocal experiment, tagged Trap-80 was detected with anti-HA after immunoprecipitation using anti-p65 (right panel). The level of p65 in total nuclear extract is shown as “input”; HA-Trap-80 was not easily detectable in nuclear extracts before immunoprecipitation. (C) BiFC mediated by in vivo interaction between p65 and Trap-80. HEK-293 cells were transfected with vectors expressing Venus fragment 2 fused to the N terminus of Trap-80 (V2-Trap-80; i, iii, iv), Trap-80–V2 (v), or p65-V2 (vi), either al [file pbio.1000073.sg001.tif]

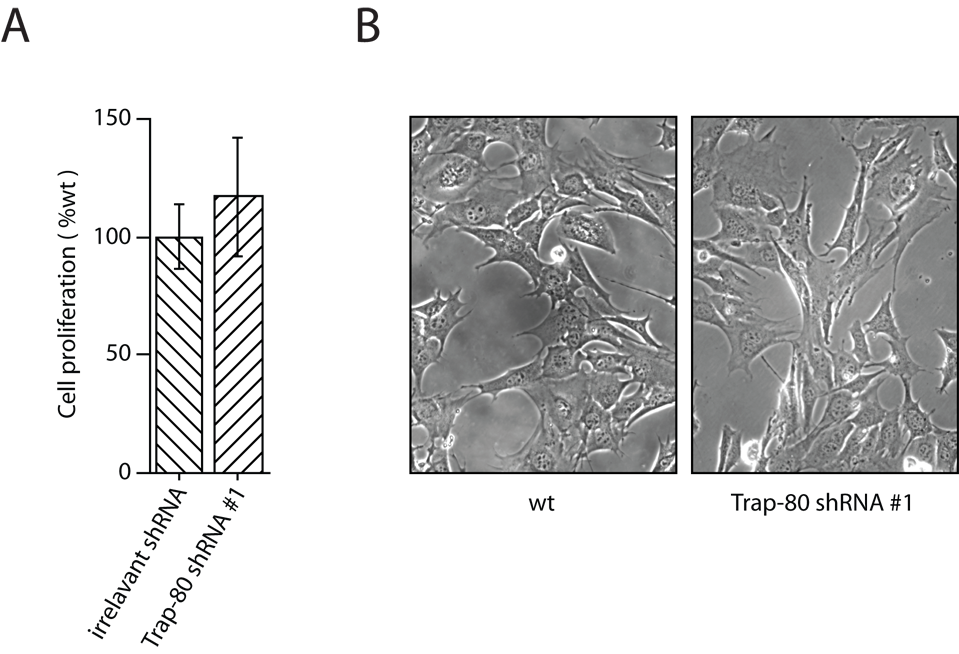

Supplement: Figure S2 — (A) Proliferation of Trap-80–deficient fibroblasts. Parallel cultures of fibroblasts expressing either the irrelevant shRNA, or an shRNA targeting Trap-80, were grown for 1 wk and the increase in cell number was determined. Proliferation is expressed as the percentage increase compared to the control culture; error bars indicate standard errors. (C) Morphological appearance of Trap-80–deficient fibroblasts. Phase contrast images (original magnification 100×) taken of exponentially growing cultures of wild-type or Trap-80 knock-down fibroblasts. (2.57 MB TIF) [file pbio.1000073.sg002.tif]

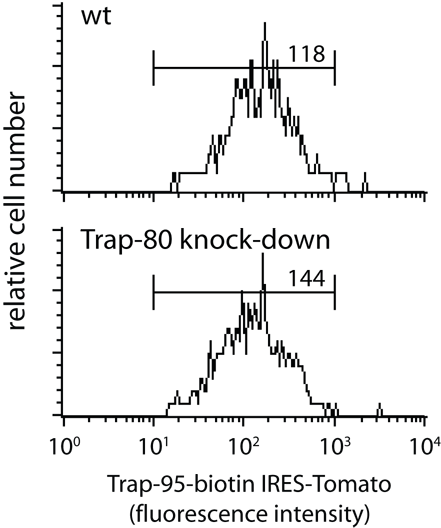

Supplement: Figure S3 — Intensity of Tomato fluorescence, expressed via an IRES sequence from a bi-cistronic mRNA also expressing Trap-95-biotin, in transduced wild-type (top) and Trap-80 knock-down (bottom) fibroblasts. The mean fluorescence level of the population is indicated. Trap-95-biotin itself co-migrates with one of two naturally biotinylated carboxylase proteins in mammalian cells, precluding direct detection on western blots. (717 KB TIF) [file pbio.1000073.sg003.tif]

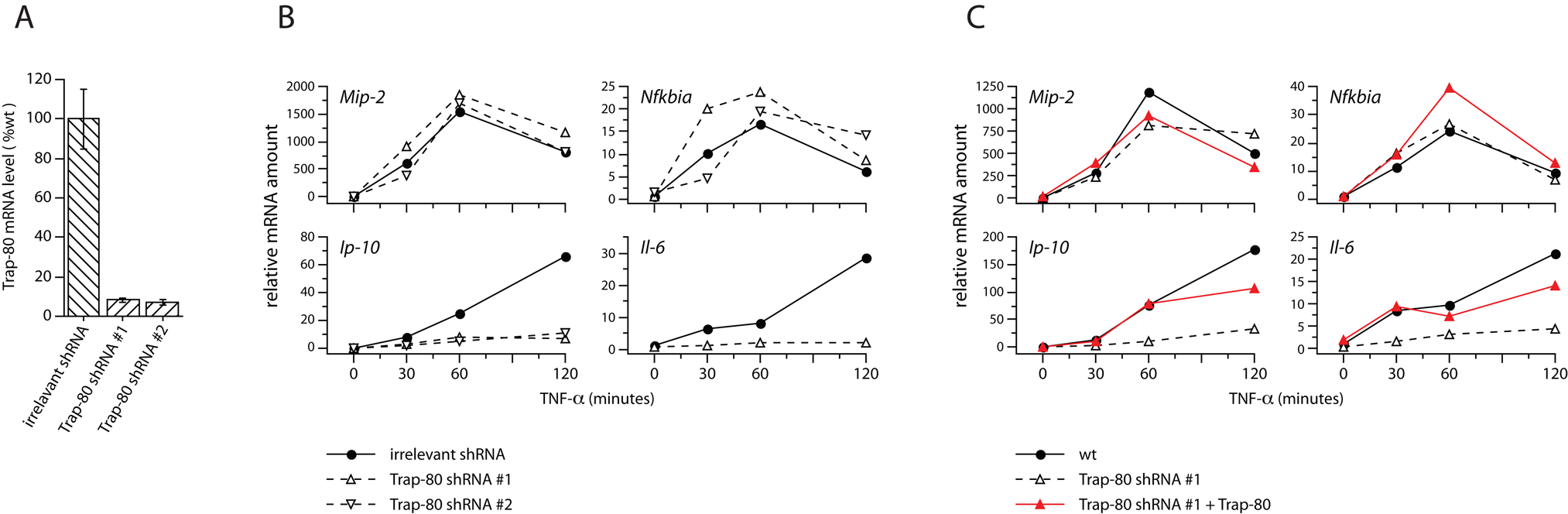

Supplement: Figure S4 — (A) Two independent hairpins targeting Trap-80 were used to generate clonal lines of 3T3 fibroblasts. A comparable number of clones was generated using each of the shRNAs targeting Trap-80, and using an irrelevant, scrambled shRNA. The degree by which Trap-80 mRNA was knocked-down varied from one clone to another, in the range of ≈70%–95% reduction of wild-type levels (not shown). The residual levels of Trap-80 mRNA are shown for a single clone expressing each shRNA, which were chosen for further study. mRNA levels are expressed as a percentage of the level in control cells. Trap-80 shRNA number 1 corresponds to the shRNA used in the experiments described in the main text. Error bars indicate standard errors; the results presented here are representative of more than ten experiments. (B) Expression of Mip-2, Nfkbia, Ip-10, and Il-6 mRNA in fibroblasts expressing either of the two shRNAs targeting Trap-80, after stimulation with TNF-α. (C) Reconstitution of Trap-80 in knock-down cells. Fibroblasts expressing an shRNA targeting Trap-80 were transduced with a retrovirus driving expression of an shRNA-resistant form of Trap-80 (red triangles; total Trap-80 mRNA levels were around 100× greater than those of wild-type cells [not shown]), and mRNA was measured as in (B). (3.97 MB TIF) [file pbio.1000073.sg004.tif]

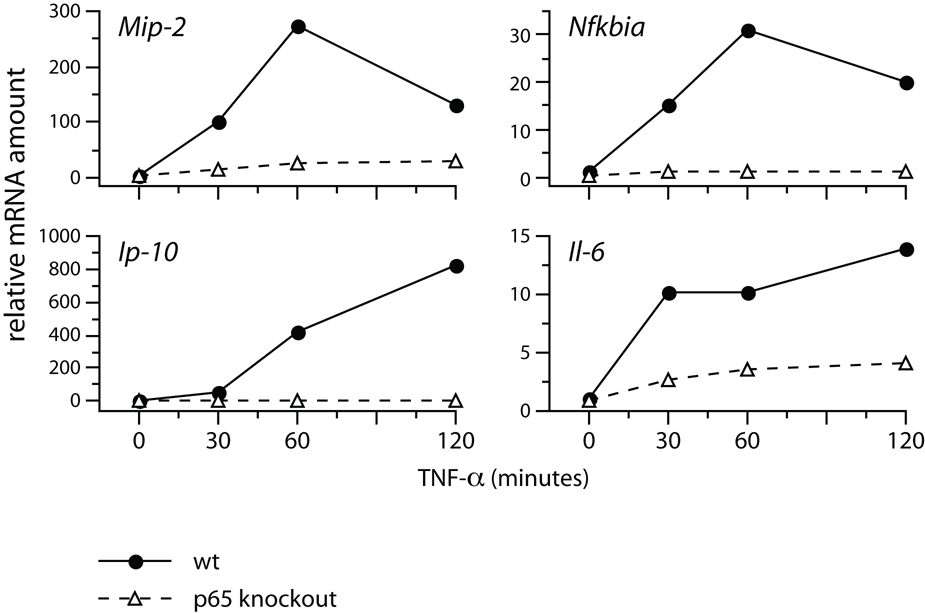

Supplement: Figure S5 — mRNA expression of Mip-2, Nfkbia, Ip-10, and Il-6, in normal and p65-knockout fibroblasts stimulated with TNF-α. The results presented here are representative of more than ten experiments. (1.69 MB TIF) [file pbio.1000073.sg005.tif]

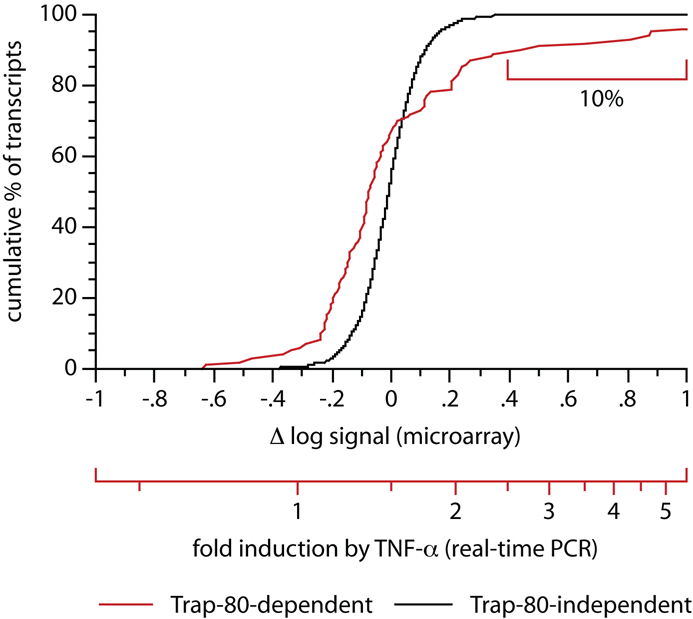

Supplement: Figure S6 — Cumulative percentage of transcripts whose expression after 1 h TNF-α stimulation exceeds that in unstimulated fibroblasts by the indicated level, expressed both as the Δ log signal of the microarray probes (upper scale) and the calculated fold induction using a standard curve generated by real-time PCR of a subset of 36 genes (lower scale). The red line represents the top 100 Trap-80–dependent transcripts; the black line represents 5000 Trap-80–independent transcripts. (1.29 MB TIF) [file pbio.1000073.sg006.tif]

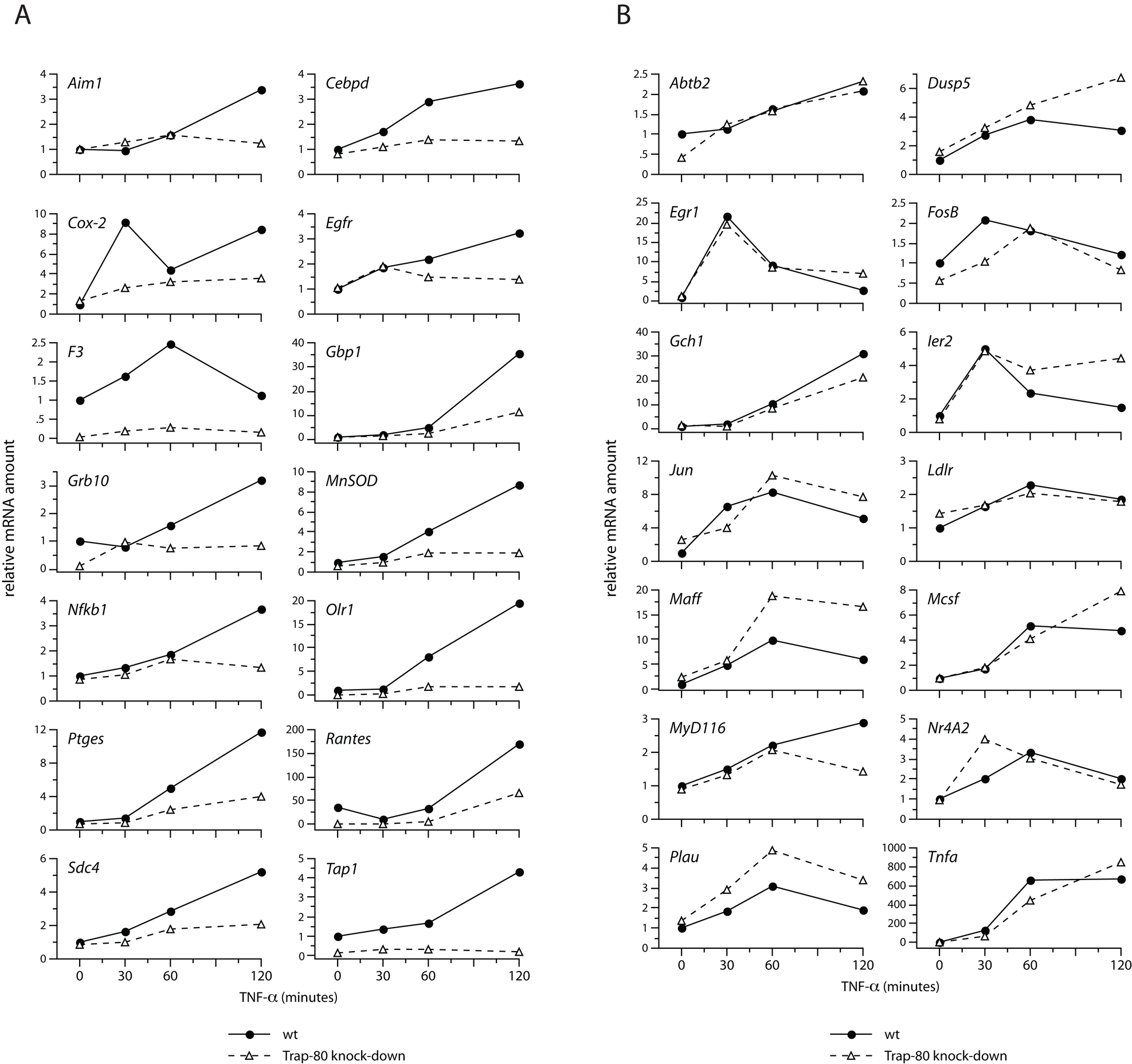

Supplement: Figure S7 — mRNA expression of a subset of Trap-80–dependent (A) and Trap-80–independent (B) genes, in wild-type and Trap-80 knock-down fibroblasts. (509 KB TIF) [file pbio.1000073.sg007.tif]

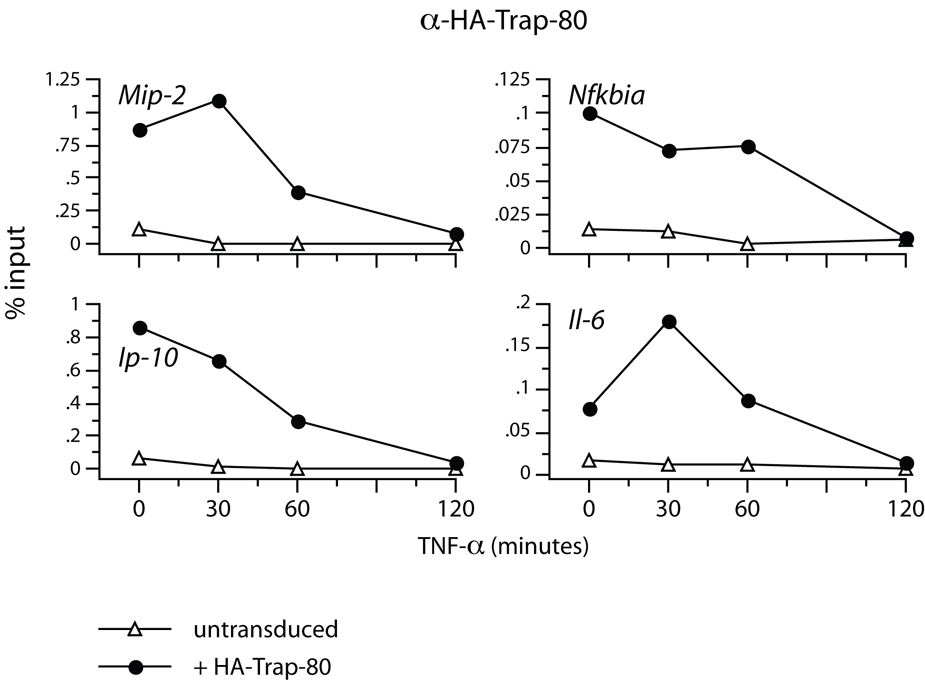

Supplement: Figure S8 — ChIP using antibodies against the HA epitope, from fibroblasts retrovirally over-expressing HA-Trap-80, or from control untransduced cells. (1.88 MB TIF) [file pbio.1000073.sg008.tif]

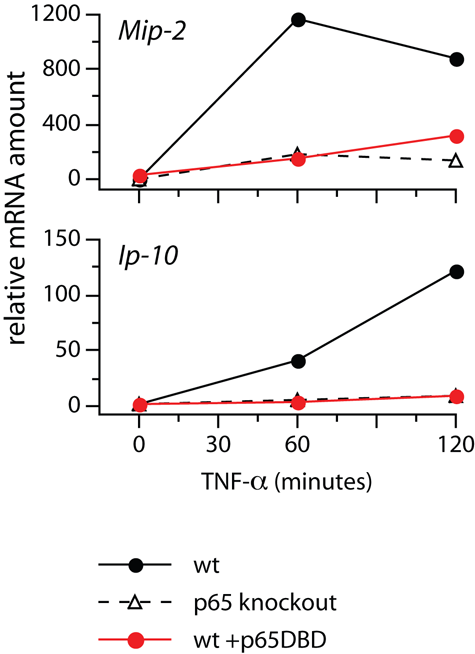

Supplement: Figure S9 — Wild-type 3T3 fibroblasts were transduced with a retrovirus driving expression of the p65 DBD (red triangles), and expression of Mip-2 and Ip-10 mRNA was compared to wild-type and p65 knockout fibroblasts. The result is representative of three experiments. (943 KB TIF) [file pbio.1000073.sg009.tif]

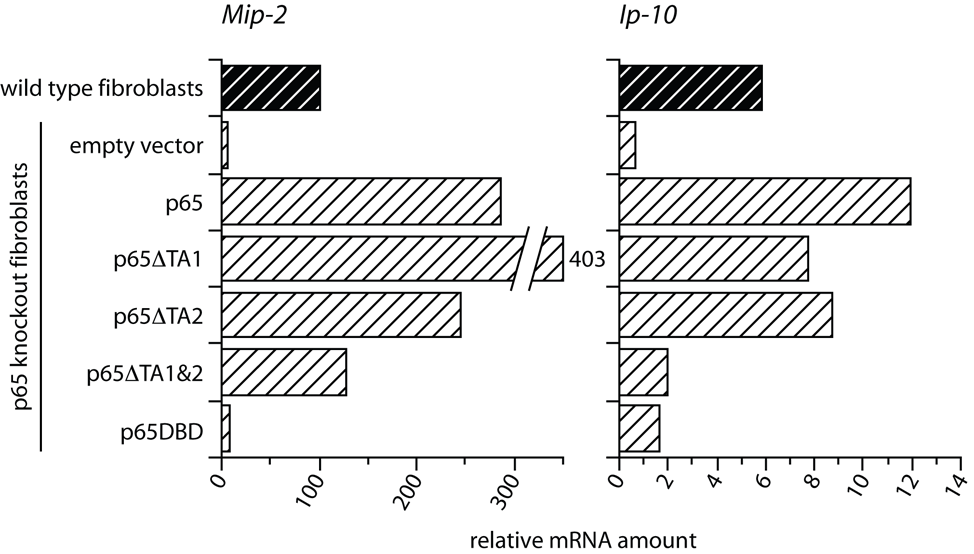

Supplement: Figure S10 — p65 knockout fibroblasts transduced with retroviruses expressing the indicated p65 mutants were stimulated with TNF-α for 1 h, and their expression of Mip-2 (left) and Ip-10 (right) mRNA was compared to that of wild-type cells. Note that the level of expression of p65 mutants in transduced cells exceeds that of endogenous p65 in wild-type cells by several-fold (not shown), which may account for the heightened expression of Mip-2. The results presented here are representative of three experiments. (1.61 MB TIF) [file pbio.1000073.sg010.tif]

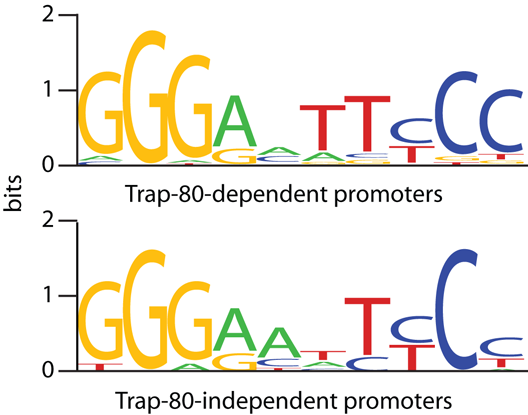

Supplement: Figure S11 — Logos of position weight matrices of NF-κB binding motifs conserved between mouse and human in Trap-80–dependent (top; n = 36) and –independent (bottom; n = 58) TNF-α–induced genes. Promoters analysed are those of the top 30 Trap-80–dependent and 30 Trap-80–independent genes, from amongst the 200 most induced by TNF-α. (677 KB TIF) [file pbio.1000073.sg011.tif]

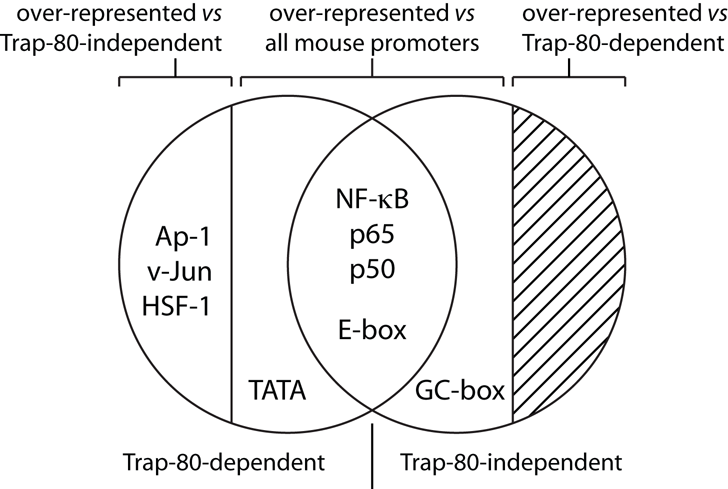

Supplement: Figure S12 — Over-represented motifs detected in promoters of TNF-α–induced genes. See Table S2 for matrices and p-values. No motifs were statistically over-represented in Trap-80–independent promoters compared with Trap-80–dependent promoters. (1.08 MB TIF) [file pbio.1000073.sg012.tif]

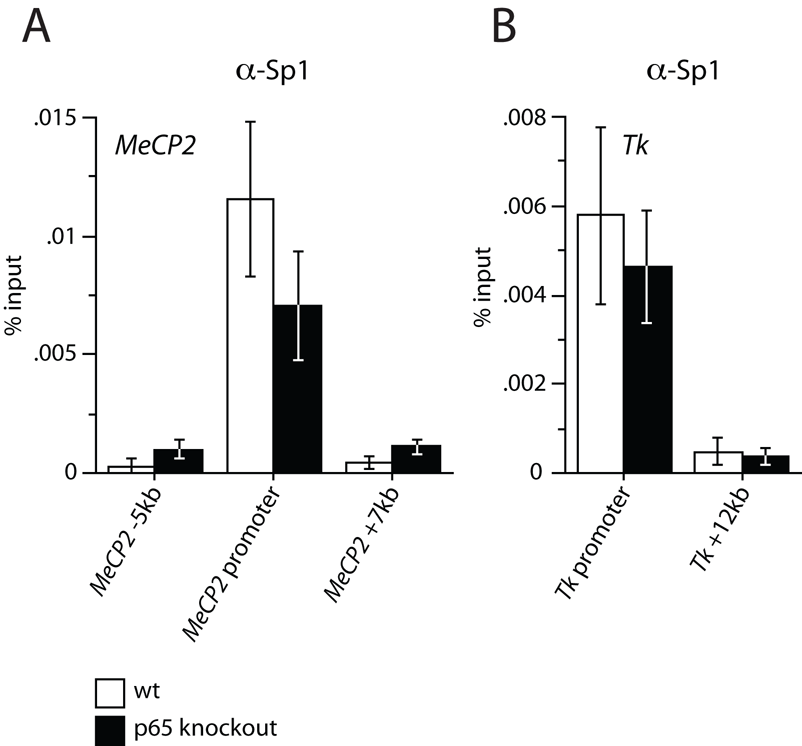

Supplement: Figure S13 — ChIP using antibodies against Sp1 in wild-type and p65-knockout fibroblasts. Primers were chosen to amplify the promoter regions of MeCP2 (A) or Tk (B), and adjacent control regions located upstream or downstream at the indicated positions. Error bars indicate the standard errors of independent immunoprecipitations (from both unstimulated and TNF-α–stimulated cells). (1.79 MB TIF) [file pbio.1000073.sg013.tif]

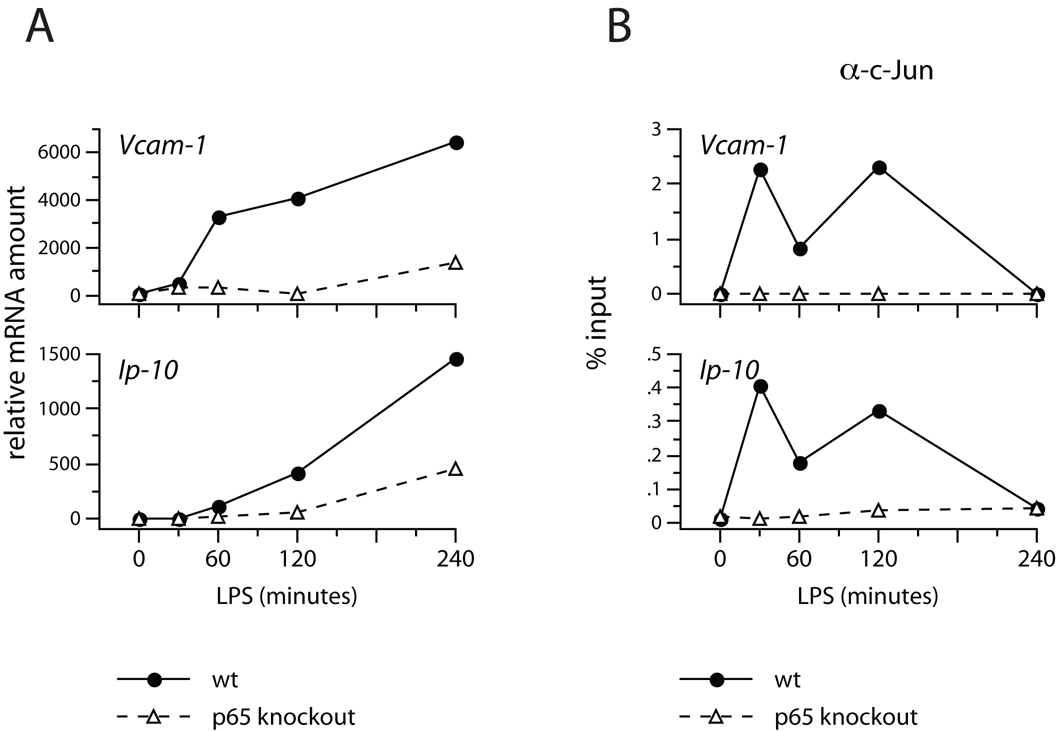

Supplement: Figure S14 — (A) p65-dependent gene transcription in dendritic cells. mRNA expression of Vcam-1 and Ip-10 in normal and p65-knockout DCs stimulated with LPS. (B) ChIP using antibodies against c-Jun. (2.51 MB TIF) [file pbio.1000073.sg014.tif]

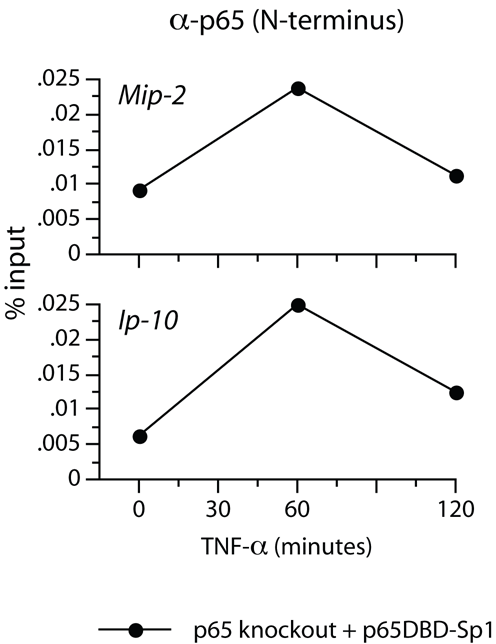

Supplement: Figure S15 — p65 knockout 3T3 fibroblasts were transduced with a retrovirus driving expression of the p65 DBD-Sp1, and ChIP was performed using antibodies against the p65 N terminus. (969 KB TIF) [file pbio.1000073.sg015.tif]

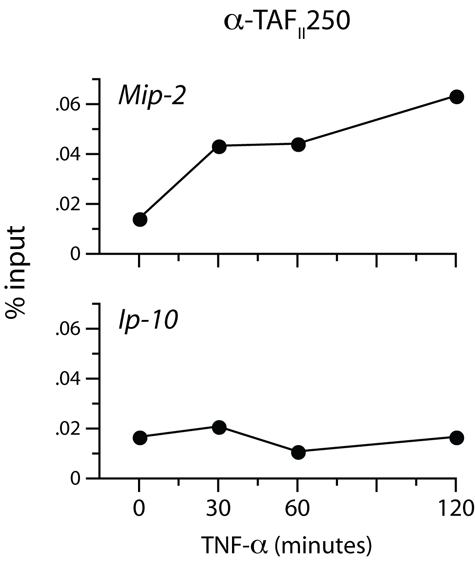

Supplement: Figure S16 — ChIP using antibodies against TAFII250 in wild-type fibroblasts. (815 KB TIF) [file pbio.1000073.sg016.tif]
