# Supplementary material for: Two Modes of Transcriptional Activation at Native Promoters by NF-κB p65
Source: PLoS Biol. 2009 Mar 31;7(3):e1000073. doi: 10.1371/journal.pbio.1000073 (PMC2661965; doi:10.1371/journal.pbio.1000073)
Supplement: Table S1 — (177 KB DOC) [file pbio.1000073.st001.doc]

Table S1: Top 50 Trap-80-dependent and Trap-80-independent TNF-α-induced genes

A. Trap-80-dependent genes:

| Gene symbol: | Fold induction by TNF-α: | Relative expression in Trap-80 knock-down: |
| --- | --- | --- |
| Tnfaip3 | 511.9 | 0.24 |
| Cxcl10 | 275.4 | 0.17 |
| Ch25h | 194.6 | 0.06 |
| Ereg | 24.7 | 0.29 |
| Fas | 12.7 | 0.24 |
| Map3k8 | 8.6 | 0.51 |
| Adm | 8 | 0.28 |
| Slc7a2 | 5.4 | 0.09 |
| Mt2 | 4.7 | 0.19 |
| Ptx3 | 4.6 | 0.15 |
| Mc5r | 4.3 | 0.14 |
| Slfn2 | 3.9 | 0.35 |
| Gpr84 | 3.7 | 0.45 |
| Rnd3 | 3.6 | 0.46 |
| Edg3 | 3.5 | 0.19 |
| Enpp2 | 3.5 | 0.29 |
| Fst | 3.4 | 0.3 |
| Cxcl9 | 3.2 | 0.44 |
| Pnrc1 | 3.1 | 0.5 |
| Mmp9 | 2.9 | 0.36 |
| Fgf23 | 2.9 | 0.42 |
| Lcn2 | 2.9 | 0.07 |
| Ifitm5 | 2.8 | 0.4 |
| Bhlhb5 | 2.8 | 0.31 |
| Ppifos | 2.8 | 0.47 |
| Pcdh18 | 2.6 | 0.43 |
| Cebpb | 2.6 | 0.37 |
| Znrf4 | 2.5 | 0.48 |
| Pbld | 2.5 | 0.47 |
| Olfr530 | 2.4 | 0.48 |
| Angpt4 | 2.4 | 0.48 |
| 5830454E08Rik | 2.3 | 0.46 |
| Tnn | 2.3 | 0.04 |
| Hoxb7 | 2.2 | 0.5 |
| C5ar1 | 2.1 | 0.5 |
| C3 | 2.1 | 0.16 |
| Creb3l3 | 2.1 | 0.5 |
| Cyp26b1 | 2.1 | 0.32 |
| Acad10 | 2.1 | 0.48 |
| Chi3l1 | 2 | 0.12 |
| Lipa | 2 | 0.32 |
| Rad52 | 2 | 0.47 |
| Penk1 | 2 | 0.14 |
| Adamts9 | 1.9 | 0.43 |
| Hapln4 | 1.9 | 0.34 |
| Amh | 1.9 | 0.45 |
| Plekhf1 | 1.9 | 0.45 |
| Adamts1 | 1.8 | 0.22 |
| Ifitm3 | 1.8 | 0.4 |
| Rftn1 | 1.8 | 0.49 |

B. Trap-80-independent genes:

| Gene symbol: | Fold induction by TNF-α: | Relative expression in Trap-80 knock-down: |
| --- | --- | --- |
| Ccl20 | 144.4 | 0.97 |
| Ccl7 | 46.5 | 0.95 |
| Nfkbia | 41.1 | 0.98 |
| Nfkbie | 32.7 | 1.05 |
| Ier3 | 15.6 | 0.99 |
| Zfp36 | 15.1 | 1.01 |
| Cxcl2 | 11.4 | 0.92 |
| Birc3 | 9.4 | 1 |
| Junb | 9.3 | 0.96 |
| Cx3cl1 | 9.1 | 1.01 |
| Klf10 | 8.9 | 1.05 |
| Zc3h12a | 6.5 | 1.05 |
| Csf1 | 5.4 | 1.03 |
| Ripk2 | 5.2 | 0.96 |
| Rel | 3.8 | 1.04 |
| Zswim4 | 3.8 | 1.07 |
| Nfkb2 | 3.8 | 0.97 |
| Tsku | 3.8 | 1.04 |
| Rapgef3 | 3.6 | 0.93 |
| Zfp36l2 | 3.6 | 1.09 |
| Cd74 | 3.4 | 1.1 |
| Tnip1 | 3.4 | 1 |
| Krt8 | 3.3 | 1.07 |
| Rfx5 | 3.2 | 1.01 |
| Plscr1 | 3.1 | 1.06 |
| Gdpd5 | 3.1 | 1.09 |
| Ccrn4l | 3 | 1.08 |
| Lbp | 3 | 0.96 |
| Ifngr2 | 3 | 1.01 |
| EG633752 | 2.9 | 1.05 |
| Myo7a | 2.9 | 0.92 |
| Oaf | 2.9 | 1.03 |
| Krt77 | 2.8 | 0.92 |
| Brp44l | 2.7 | 1 |
| Asb2 | 2.7 | 0.95 |
| Bcl10 | 2.7 | 0.97 |
| Pik3r5 | 2.6 | 0.96 |
| Ghrl | 2.6 | 0.92 |
| 2900009I07Rik | 2.5 | 0.91 |
| LOC674596 | 2.5 | 1.02 |
| 1700055N04Rik | 2.5 | 0.96 |
| Itga2 | 2.4 | 0.92 |
| C330016O10Rik | 2.4 | 1.05 |
| Foxe3 | 2.4 | 0.91 |
| Prr19 | 2.4 | 0.97 |
| Dmrt3 | 2.4 | 0.97 |
| 9530008L14Rik | 2.4 | 1.07 |
| 2310051M13Rik | 2.4 | 0.98 |
| Gnat1 | 2.4 | 0.97 |
| Gm1070 | 2.3 | 0.97 |
