# Supplementary material for: Two Modes of Transcriptional Activation at Native Promoters by NF-κB p65
Source: PLoS Biol. 2009 Mar 31;7(3):e1000073. doi: 10.1371/journal.pbio.1000073 (PMC2661965; doi:10.1371/journal.pbio.1000073)
Supplement: Table S2 — (113 KB DOC) [file pbio.1000073.st002.doc]

Table S2: Over-represented motifs in TNF-α-induced gene promoters

A. Over-represented in both both Trap-80-dependent and –independent *vs* all mouse promoters:

| Motif: | TransFac matrix: | p-value: | Note: |
| --- | --- | --- | --- |
| NF-κB | M00054 | 2x10^-9^ |  |
| p65 | M00052 | 2x10^-8^ |  |
| p50 | M00051 | 3x10^-6^ |  |
| Hen-1 | M00068 | 5x10^-8^ | E-box |
|  | M00058 | 2x10^-7^ | E-box |
| Tal-1β/ITF2 | M00070 | 10^-6^ | E-box |
| E47 | M00071 | 10^-5^ | E-box |

B. Over-represented in Trap-80-independent *vs* all mouse promoters:

| Motif: | TransFac matrix: | p-value: | Note: |
| --- | --- | --- | --- |
| Pax-4 | M00380 | 6x10^-10^ | E-box |
| GC-box | M00255 | 7x10^-7^ | inc. Sp-1 binding site |
| NF-κB | M00194 | 3x10^-3^ |  |

C. Over-represented in Trap-80-dependent *vs* all mouse promoters:

| Motif: | TransFac matrix: | p-value: | Note: |
| --- | --- | --- | --- |
| TATA | M00252 | 4x10^-6^ |  |

D. Over-represented in Trap-80-dependent *vs* Trap-80-independent promoters:

| Motif: | TransFac matrix: | p-value: | Note: |
| --- | --- | --- | --- |
| Ap-1 | M00517 | 2x10^-13^ |  |
|  | M00199 | 10^-10^ |  |
|  | M00174 | 3x10^-9^ |  |
|  | M00188 | 2x10^-5^ |  |
|  | M00173 | 10^-3^ |  |
| v-Jun | M00036 | 10^-4^ |  |
| NF-E2 | M00037 | 3x10^-9^ | inc. Ap-1 binding site |
| Tcf-11/MafG | M00284 | 2x10^-4^ | inc. Ap-1 binding site |
| HSF-1 | M00146 | 2x10^-4^ |  |
| HSF-2 | M00147 | 2x10^-3^ |  |
| TATA | M00252 | 2x10^-3^ |  |
